# Optimized genetic tools for neuroanatomical and functional mapping of the *Aedes aegypti* olfactory system

**DOI:** 10.1093/g3journal/jkae307

**Published:** 2025-01-24

**Authors:** Shruti Shankar, Diego Giraldo, Genevieve M Tauxe, Emma D Spikol, Ming Li, Omar S Akbari, Margot P Wohl, Conor J McMeniman

**Affiliations:** W. Harry Feinstone Department of Molecular Microbiology and Immunology, Johns Hopkins Malaria Research Institute, Johns Hopkins Bloomberg School of Public Health, Johns Hopkins University, Baltimore, MD 21205, USA; W. Harry Feinstone Department of Molecular Microbiology and Immunology, Johns Hopkins Malaria Research Institute, Johns Hopkins Bloomberg School of Public Health, Johns Hopkins University, Baltimore, MD 21205, USA; W. Harry Feinstone Department of Molecular Microbiology and Immunology, Johns Hopkins Malaria Research Institute, Johns Hopkins Bloomberg School of Public Health, Johns Hopkins University, Baltimore, MD 21205, USA; The Solomon H. Snyder Department of Neuroscience, Johns Hopkins University School of Medicine, Baltimore, MD 21205, USA; Department of Cell and Developmental Biology, School of Biological Sciences, University of California, San Diego, La Jolla, CA 92093, USA; Department of Cell and Developmental Biology, School of Biological Sciences, University of California, San Diego, La Jolla, CA 92093, USA; W. Harry Feinstone Department of Molecular Microbiology and Immunology, Johns Hopkins Malaria Research Institute, Johns Hopkins Bloomberg School of Public Health, Johns Hopkins University, Baltimore, MD 21205, USA; W. Harry Feinstone Department of Molecular Microbiology and Immunology, Johns Hopkins Malaria Research Institute, Johns Hopkins Bloomberg School of Public Health, Johns Hopkins University, Baltimore, MD 21205, USA; The Solomon H. Snyder Department of Neuroscience, Johns Hopkins University School of Medicine, Baltimore, MD 21205, USA

**Keywords:** mosquito, CRISPR–Cas9, olfaction, chemoreceptor, neurogenetics

## Abstract

The mosquito *Aedes aegypti* is an emerging model insect for invertebrate neurobiology. We detail the application of a dual transgenesis marker system that reports the nature of transgene integration with circular donor template for CRISPR–Cas9-mediated homology-directed repair at target mosquito chemoreceptor genes. Employing this approach, we demonstrate the establishment of cell-type-specific *T2A-QF2* driver lines for th*e A. aegypti* olfactory co-receptor genes *Ir8a* and *orco* via canonical homology-directed repair and the CO_2_ receptor complex gene *Gr1* via noncanonical homology-directed repair involving duplication of the intended *T2A-QF2* integration cassette separated by intervening donor plasmid sequence. Using *Gr1*+ olfactory sensory neurons as an example, we show that introgression of such *T2A-QF2* driver and *QUAS* responder transgenes into a *yellow* cuticular pigmentation mutant strain facilitates transcuticular calcium imaging of CO_2_-evoked neural activity on the maxillary palps with enhanced sensitivity relative to wild-type mosquitoes enveloped by dark melanized cuticle. We further apply Cre-loxP excision to derive marker-free *T2A-QF2* in-frame fusions to clearly map axonal projection patterns from olfactory sensory neurons expressing these 3 chemoreceptors into the *A. aegypti* antennal lobe devoid of background interference from *3xP3-*based fluorescent transgenesis markers. The marker-free *Gr1 T2A-QF2* driver facilitates clear recording of CO_2_-evoked responses in this central brain region using the genetically encoded calcium indicators GCaMP6s and CaMPARI2. Systematic application of these optimized methods to different chemoreceptors stands to enable mapping *A. aegypti* olfactory circuits at peripheral and central levels of olfactory coding at high resolution.

## Introduction

The yellow fever mosquito *Aedes aegypti* is a globally important vector of yellow fever, dengue, Zika, and chikungunya viruses. These mosquitoes use their highly tuned sense of smell to detect humans, find sources of nectar, and locate oviposition sites. Given these complex olfactory behaviors, including epidemiologically relevant ones such as anthropophilic host-seeking behavior, *A. aegypti* has become an important insect model to study chemosensation and sensory integration (DeGennaro *et al*. 2013; McBride *et al*. 2014; McMeniman *et al*. 2014; van Breugel *et al*. 2015; Duvall *et al*. 2019; Raji *et al*. 2019; Jové *et al*. 2020; Melo *et al*. 2020; De Obaldia *et al*. 2022; Herre *et al*. 2022; San Alberto *et al*. 2022; Chandel *et al*. 2024; Rouyar *et al*. 2024). Studies of *A. aegypti* sensory neurobiology have been catalyzed directly as the result of a high-quality genome annotation (Matthews *et al*. 2018), comprehensive neurotranscriptomic datasets (Matthews *et al*. 2016; Herre *et al*. 2022), and the application of genome engineering tools including the *QF2/QUAS* binary expression system and genome editing tools such as the CRISPR–Cas9 system to assess gene function in this mosquito species (Kistler *et al*. 2015; Li *et al*. 2017; Matthews *et al*. 2019; Herre *et al*. 2022).

The olfactory system of *A. aegypti* consists of 3 major olfactory appendages including the antennae, the maxillary palps, and the labella of the proboscis. Lining these organs are various morphological classes of porous sensilla (McIver 1972, 1973, 1978) that house the dendritic processes of typically 2–3 olfactory sensory neurons (OSNs) that detect diverse structural classes of volatile odorants. Large chemoreceptor gene families implicated in detection of various components of human scent and other ethologically relevant odorants are encoded in the *A. aegypti* genome (Matthews *et al*. 2018) and are expressed by olfactory appendages on the head (Herre *et al*. 2022). The odorant receptor (OR) chemoreceptor family, typically tuned to short-chain alcohols, ketones, and aromatic compounds, likely contributes toward anthropophilic host preference in *A. aegypti* (DeGennaro *et al*. 2013; McBride *et al*. 2014). In a complementary fashion, chemoreceptors from the ionotropic receptor (IR) family that are responsive to carboxylic acids and amines (Raji *et al*. 2019) and certain gustatory receptor (GR) family members that detect the volatile gas carbon dioxide (CO_2_) (McMeniman *et al*. 2014) synergistically drive behavioral taxis of female mosquitoes toward human scent.

*T2A-QF2* in-frame fusions have been applied to map expression patterns of target chemosensory genes from the OR, IR, and GR families in the olfactory appendages of *Drosophila* (Task *et al*. 2022), *Anopheles coluzzii* and *Anopheles gambiae* (Raji *et al*. 2019; Ye *et al*. 2022; Giraldo *et al*. 2024), and *A. aegypti* (Herre *et al*. 2022; Zhao *et al*. 2022). These neurogenetic tools leveraging *QF2/QUAS*-mediated binary expression (Riabinina *et al*. 2015) have also been used to express genetically encoded calcium indicators (GECIs) to study the ligand tuning dynamics of different sets of sensory neurons in the peripheral sensory organs of *A. coluzzii* and *A. gambiae* (Laursen *et al*. 2023; Raji *et al*. 2023; Giraldo *et al*. 2024) and taste neurons in the *A. aegypti* stylet (Jové *et al*. 2020). Application of *T2A-QF2*/*QUAS*-mediated cell-type-specific expression has also permitted optogenetic activation of CO_2_-sensitive neurons in the maxillary palps of *A. aegypti* to help expand our understanding of CO_2_-activated behavioral state changes in this mosquito (Sorrells *et al*. 2022). While optogenetics appears to have some success, there are limited reports of calcium imaging in the peripheral sensory organs in *A. aegypti*, likely because its olfactory appendages are enveloped by dark, highly melanized cuticle which dampens recording of stimulus-evoked fluorescent calcium signals from GECIs.

Moving centrally, the axonal processes of OSNs project to the primary olfactory processing center of the *A. aegypti* brain known as the antennal lobe (Ignell *et al*. 2005; Shankar and McMeniman 2020). In related insects such as *Drosophila*, olfactory information is locally processed and encoded in the antennal lobe via the action of excitatory and inhibitory local neurons (Chou *et al*. 2010; Yaksi and Wilson 2010; Hong and Wilson 2015), before being sent by projection neurons to higher-order brain centers involved in orchestrating innate and learned olfactory behaviors (Caron *et al*. 2013; Grabe *et al*. 2016; Dolan *et al*. 2019). Neurogenetic tools have allowed the recent application of GCaMP calcium indicators to study olfactory processing in the *A. aegypti* antennal lobe and other brain regions (Bui *et al*. 2019; Vinauger *et al*. 2019; Lahondère *et al*. 2020; Melo *et al*. 2020; Zhao *et al*. 2021, 2022; Wolff *et al*. 2023). These studies have facilitated identification of regions in the AL where human odor is putatively encoded by neurons expressing the OR co-receptor Orco (Zhao *et al*. 2022), as well as glomeruli that encode for flower odors (Lahondère *et al*. 2020) and oviposition cues (Melo *et al*. 2020). Since the antennal lobe is the first relay station for processing olfactory information, high-resolution mapping of the neuroanatomy of this brain center is crucial to discern the neural basis of mosquito olfactory coding.

Here, we describe a series of optimized genetic tools for neuroanatomical and functional mapping of the *A. aegypti* olfactory system. Using CRISPR–Cas9 homology-directed repair (HDR) donors that report the nature of transgene integration with a dual *3xP3*-based marker system, we generate precise *T2A-QF2* in-frame fusions in 3 target *A. aegypti* chemoreceptor genes, namely *Ir8a*, *orco*, and *Gr1*, for application in binary combination with various *QUAS* responders. Using *Gr1*+ OSNs as an example, we demonstrate that introgression of *Gr1 T2A-QF2* driver and *QUAS-GCaMP6s* responder transgenes into a mutant *yellow* background that lacks cuticular melanization (Li *et al*. 2017) increases the signal obtained from peripheral sensory neurons during calcium imaging by 2–4-fold. We further show that Cre-loxP-mediated excision of commonly used *3xP3* markers (Berghammer *et al*. 1999) facilitates clear mapping of axonal projection patterns of these different subsets of OSNs into the antennal lobe, devoid of background marker interference. We provide an updated map of the *A. aegypti* antennal lobe detailing the projection patterns of OSNs expressing these chemoreceptors to specific glomeruli in this brain region. Finally, we demonstrate that neurons expressing *Gr1* project to the largest glomerulus (MD1) in the *A. aegypti* antennal lobe and determine using calcium imaging with GCaMP6s (Chen *et al*. 2013) and the photoactivatable genetically encoded calcium indicator CaMPARI2 (Moeyaert *et al*. 2018) that this glomerulus mediates CO_2_ detection. These optimized genetic tools will facilitate systematic studies that aim to decode the ligand tuning dynamics and principles of *A. aegypti* olfactory processing at both peripheral and central levels to improve our fundamental understanding of the chemosensory biology of this prolific disease vector.

## Materials and methods

### Mosquito strains and maintenance

The *A. aegypti LVPib12* strain (Nene *et al*. 2007) was used as the genetic background for generation of all transgenic lines and subsequent assays. *exu-Cas9* and *yellow* mutant strains were generated and provided by Li *et al*. (2017). Mosquitoes were maintained with a 12-h light:dark photoperiod at 27°C and 80% relative humidity using a standardized rearing protocol (Wohl and McMeniman 2023). All experiments were conducted with mated, nonblood fed *A. aegypti* females that were 5–10 days old. Adult mosquitoes were provided constant access to a 10% w/v sucrose solution. Stock and composite genotypes used in each figure panel are detailed in Supplementary Table 1 in Supplementary File 1.

### Selection and in vitro transcription of single-guide RNAs

Single-guide RNA (sgRNA) target sites were identified in the coding sequences of *orco* (AAEL005776), *Ir8a* (AAEL002922), and *Gr1* (AAEL002380) (Supplementary Table 2 in Supplementary File 1). Candidate sgRNAs at each locus were prioritized for downstream use based on their putative lack of off-target activity in the *A. aegypti* genome. sgRNAs were transcribed and purified according to the method of Kistler *et al*. (2015). Briefly, DNA templates for sgRNA synthesis were generated by PCR with 2 partially overlapping PAGE-purified oligos (IDT) for each target. sgRNA was subsequently produced using the MegaScript T7 in vitro transcription kit (Ambion) and purified using the MEGAclear transcription clean-up kit (Invitrogen). Prior to microinjection, sgRNA activity was confirmed by in vitro cleavage assays with purified recombinant Cas9 protein (PNA Bio, Inc., CP01-200) following the manufacturer's instructions.

### *T2A-QF2* donor constructs

A base *T2A-QF2* donor construct (*pBB*) for CRISPR–Cas9-mediated homologous recombination into target chemoreceptor loci in *A. aegypti* was generated by sequential rounds of In-Fusion cloning (TakaraBio, 639650). This construct that was generated in a pSL1180 backbone has an EcoNI site for in-frame fusion of a 5′ homology arm from a target gene with *T2A-QF2*, and additional restriction sites including BssHII site for insertion of the 3′ homology arm. To survey for homology arms, genomic DNA regions spanning each target site were first PCR-amplified with CloneAmp (TakaraBio, 639298) using the following primers for *orco* (5′-TGCAAGTGGATCATTTGTCG-3′ and 5′-GTGCAATTGTGCCATTTTGA-3′), *Ir8a* (5′-CAAAGTATAATTTCGCCCCCTCC-3′ and 5′-CTCTATGGCAGCCAAGATATTGG-3′), and *Gr1* (5′-AAGCCAGCTGGAAGGACATA-3′ and 5′-ACCGTTTGGAGGTTGAATTG-3′). PCR products were cloned into pCR2.1-TOPO (Invitrogen) for subsequent sequence verification. After determining the most common sequence clone for each region, homology arms flanking the CRISPR–Cas9 cut site were PCR-amplified and inserted into the *pBB* donor at the EcoNI site (5′ arm) and BssHII site (3′ arm) using the In-Fusion primers detailed in Supplementary Table 3 in Supplementary File 1, to generate a *T2A* in-frame fusion into the coding exon of interest. Three donor constructs that yielded successful integrations at these target loci included *pBB-AaOrco*, *pBB-AaIR8a*, and *pBB-AaGr1*. Each *T2A-QF2* donor construct included a floxed *3xP3-DsRed2* transformation marker, as well as an unfloxed *3xP3-ECFP* marker in the vector backbone outside the HDR cassette. This latter *3xP3-ECFP* marker was used to assess putative vector backbone integration events at the target locus or alternate off-target integrations elsewhere in the genome. *orco^QF2–3xP3^* and *Ir8a^QF2–3xP3^* cassettes inserted in-frame as expected via canonical HDR. The *Gr1^QF2–3xP3^* cassette inserted in-frame but incorporated a duplicated copy of the donor cassette along with intervening plasmid backbone sequence downstream of the *T2A-QF2* in-frame fusion via a noncanonical HDR repair event.

### *Mos1 mariner QUAS* responder and germline *Cre* constructs

*QUAS* responder and germline *Cre* cassettes were generated by sequential rounds of In-Fusion cloning (TakaraBio) into template plasmid backbones for *Mos1 mariner* transposition (Coates *et al*. 1998). All *QUAS* reporter constructs included a *3xP3-ECFP* transformation marker. The *pMOS* cassette for *15xQUAS-CaMPARI2* was modified to include a floxed *3xP3-ECFP* marker, and the *pMOS* backbone for *exu-Cre* was modified to have a *Polyubiquitin-EYFP* marker using standard cloning methods. Final plasmids that yielded transformants included: *pMosECFP-15xQUAS-mCD8GFP*, *pMosECFP-15xQUAS-GCaMP6s*, *pMos-loxP-ECFP-loxP-15xQUAS-CaMPARI2*, and *pMosEYFP-exu-Cre*.

All template materials used to generate the constructs in this study are detailed in Supplementary Table 4 in Supplementary File 1. Stellar Competent *Escherichia coli* cells (Takara, 636763) were used for all cloning and plasmid preparations.

### Generation of transgenic lines

*T2A-QF2* knock-in lines were generated via CRISPR–Cas9-mediated HDR using embryonic microinjection. To generate the *Gr1^QF2–3xP3^* insertion, an injection mixture consisting of sgRNA (40 ng/µL), purified recombinant Cas9 protein (PNA Bio, 300 ng/µL), and donor plasmid (500 ng/µL) was prepared in microinjection buffer (5 mM KCl and 0.1 mM NaH_2_PO_4_, pH 7.2) and microinjected into the posterior pole of preblastoderm stage *LVPib12* embryos at the Insect Transformation Facility at University of Maryland (UM-ITF) using standard methods. To generate the *orco^QF2–3xP3^* and *Ir8a^QF2–3xP3^* insertions, sgRNA (100 ng/µL) was mixed with the *T2A-QF2* donor construct (100 ng/µL) for each target and microinjected into the posterior pole of transgenic *A. aegypti* preblastoderm stage embryos expressing *Cas9* under the maternal germline promoter *exuperantia* (Li *et al*. 2017) at Johns Hopkins.

Transformed G_1_ larvae from all knock-in lines were initially isolated via the visible expression of *3xP3-DsRed2* marker in eye tissue. Putative noncanonical HDR events were screened for by examining these G_1_ larvae for co-expression of *3xP3-DsRed2* and *3xP3-ECFP* in eye tissue. Transgenics were outcrossed to the *LVPib12* wild-type (WT) line for at least 5 generations prior to attempting to generate homozygous strains. A single representative *T2A-QF2* in-frame fusion event for each gene target was chosen for downstream characterization, prioritizing canonical HDR insertions for each locus if they were available. Precise insertion of each donor construct in these mosquito lines was confirmed by PCR amplification and subsequent Sanger sequencing of regions covering the homology arms and flanking sequences on either side of the insertion. For logistical feasibility of stock maintenance, redundant mosquito lines derived from noncanonical HDR events or canonical HDR events were discarded after successful establishment of these sequence-validated driver lines.

*QUAS* responder and *exu-Cre* strains were generated by co-injecting each *pMOS* donor construct (500 ng/µL) with a pKhsp82 helper plasmid (300 ng/µL) expressing the *Mos1* transposase (Coates *et al*. 1998) for quasi-random integration into the genome. Embryo microinjections to generate these strains were carried out by UM-ITF using standard techniques. For *QUAS* responders, the G_1_ offspring selected for line establishment were those that had the strongest *3xP3-ECFP* marker expression levels in the eyes and ventral nerve cord, indicative of responder loci accessible for neuronal expression.

### Cre-loxP-mediated excision of *3xP3* fluorescent markers

To remove floxed *3xP3* marker cassettes, we crossed males of each *QF2* driver line (*Ir8a^QF2–3xP3^*, *orco^QF2–3xP3^*, *Gr1^QF2–3xP3^*) to females of the *exu-Cre* line we generated. We then screened F_1_ progeny for loss of the *3xP3* fluorescent markers. Precise excision was confirmed for all 3 driver lines by PCR and Sanger sequencing.

### *Mos1 mariner* Splinkerette PCR

*QUAS* and *exu-Cre* transgenes inserted via *Mos1* mariner transposition were mapped to chromosomal locations (AaegL5.0 genome assembly) using a modified Splinkerette PCR (Potter and Luo 2010). Genomic DNA from single transgenic individuals was digested using the restriction enzymes BamHI-HF, BglII, and BstYI (New England BioLabs) in separate reactions. Digests were left overnight (∼16 h). BstYI reactions were subsequently heat-inactivated at 80°C for 20 min according to the recommended protocol. BamHI reactions were purified using the QIAquick PCR Purification Kit (QIAgen) according to manufacturer instructions and eluted in 50 μl H_2_O after 4 min of incubation at 50°C.

Digests of genomic DNA were ligated to annealed Splinkerette (SPLNK) oligos as described (Potter and Luo 2010). SPLNK oligonucleotides 5′-GATCCCACTAGTGTCGACACCAGTCTCTAATTTTTTTTTTCAAAAAAA-3′ and 5′-CGAAGAGTAACCGTTGCTAGGAGAGACCGTGGCTGAATGAGACTGGTGTCGACACTAGTGG-3′ were first annealed and ligated to digested genomic DNA. The first- and second-round PCR amplification steps were modified using the standard SPLNK oligos and new primers designed for the inverted repeat regions of the *Mos1 mariner* transposon. PCR products were amplified using Phusion High-Fidelity DNA Polymerase (NEB).

The first-round splinkerette PCR was carried out using the primers 5′-CGAAGAGTAACCGTTGCTAGGAGAGACC-3′ and 5′-TCAGAGAAAACGACCGGAAT-3′ for the right inverted repeat and 5′-CGAAGAGTAACCGTTGCTAGGAGAGACC-3′ and 5′-CACCACTTTTGAAGCGTTGA-3′ for the left inverted repeat. The second-round splinkerette PCR was carried out using the primers 5′-GTGGCTGAATGAGACTGGTGTCGAC-3′ and 5′-TCCGATTACCACCTATTCGC-3′ for the right inverted repeat and 5′-GTGGCTGAATGAGACTGGTGTCGAC-3′ and 5′-ATACTGTCCGCGTTTGCTCT-3′ for the left inverted repeat. For *QUAS-CaMPARI2*, the extension time of the second-round PCR was lengthened to 4 min to amplify longer segments of flanking DNA. PCR products were gel purified and Sanger sequenced with additional sequencing primers for the right (5′-AAAAATGGCTCGATGAATGG-3′) and left (5′-GGTGGTTCGACAGTCAAGGT-3′) inverted repeats. BLAST searches were used to map splinkerette fragments derived from each *Mos1 mariner* cassette to coordinate locations in the genome at canonical TA dinucleotides (Richardson *et al*. 2009), and insertion sites (Supplementary Table 5 in Supplementary File 1) were subsequently confirmed by PCR.

### Genotyping Gr1^QF2–3xP3^ and Gr1^QF2-MF^

*Gr1^QF2–3xP3^* and *Gr1^QF^*^2*-MF*^ knock-ins were genotyped using a multiprimer PCR assay with the forward primer: 5′-CATGTACATCCGCAAGTTGG-3′ and 2 standard reverse primers: 5′-TGTTAGTGAGATCAGCGAACCT-3′ and 5′-GATCAACCCACAGATGACGA-3′. Fragments for size-based genotyping were amplified via DreamTaq (Thermo Scientific) and analyzed by conventional agarose gel electrophoresis. Each of the reverse primers was used at half the normal concentration. This resulted in a single 689-bp amplicon in homozygous mosquitoes; a single 884-bp amplicon in WT mosquitoes; and 2 amplicons, 1 at 689 bp and 1 at 884 bp, in heterozygous mosquitoes.

To characterize the nature of the HDR insertion in *Gr1^QF2–3xP3^* which contained both *3xP3*-DsRed2 and *3xP3-ECFP* markers indicative of both donor cassette and plasmid backbone integration, we used PCR to amplify 2 overlapping fragments. The first amplicon (6,770 bp) had primers anchored in the genomic region outside the left homology arm (5′-TCGCTGAGTGATGAGGGTTT-3′) and within *ECFP* (5′-CTTCTCGTTGGGGTCTTTGC-3′). The second amplicon (8,432 bp) had primers anchored within *ECFP* (5′-GAGGAGCTGTTCACCGGG-3′) and in the genomic region outside the right homology arm (5′-CATGAATGCCCAAGACCATCT-3′). PCR fragments were gel purified (Wizard SV Gel and PCR Cleanup System, Promega) and Nanopore sequenced (Plasmidsaurus) for assembly.

### Genotyping *Ir8a^QF2–3xP3^* and *Ir8a^QF2-MF^*

*Ir8a^QF2–3xP3^* and *Ir8a^QF2-MF^* knock-ins were genotyped using a multiprimer PCR assay with the forward primer: 5′-AGGAGATTGCGCTTGTCCTA-3′ and 2 reverse primers: 5′-CCCCGACATAGTTGAGCATT-3′ and 5′-TGTTAGTGAGATCAGCGAACCT-3′. Each of the reverse primers was used at half the normal concentration. This resulted in a single 560-bp amplicon in homozygous mosquitoes; a single 501-bp amplicon in WT mosquitoes; and 2 amplicons, 1 at 560 bp and 1 at 501 bp in heterozygous mosquitoes.

### Genotyping *orco^QF2–3xP3^* and *orco^QF2-MF^*

*orco^QF2–3xP3^* and *orco^QF2-MF^* knock-ins were genotyped using conventional PCR. The PCR used the forward primer: 5′-GCGATAGCGTCAAAAACGTA-3′ and reverse primer: 5′-ATTCCTTGAAGGTCCATTGCAG-3′. This resulted in a 1,842-bp amplicon corresponding to the *orco^QF2-MF^* allele, a 3,129-bp amplicon corresponding to the *orco^QF2–3xP3^* allele, and a 367-bp amplicon corresponding to the WT allele. Heterozygotes had both WT and transgenic PCR bands.

### Genotyping *15xQUAS-mCD8::GFP*

*15xQUAS-mCD8::GFP* was genotyped using conventional PCR. The PCR used the forward primer: 5′-TCCAGCCGATAGGAACAATC-3′ and reverse primer: 5′-CAAATCCGAATTTCCCGTAA-3′. This resulted in a single 5,797-bp amplicon for homozygotes and a 444-bp amplicon for the WT allele. Heterozygotes typically only had the WT PCR band given the size differential between these 2 amplicons.

### Genotyping *15xQUAS-GCaMP6s*

*15xQUAS-GCaMP6s* was genotyped using a multiprimer PCR with the forward primer: 5′-CCAATCCCTCCAAAACAAGA-3′; and 2 reverse primers: 5′-ACGCTTTCGACAGATTCGTT-3′ and 5′-CACCACTTTTGAAGCGTTGA-3′. Each of the reverse primers was used at half the normal concentration. This resulted in a single 529-bp amplicon in homozygous mosquitoes; a single 379-bp amplicon in WT mosquitoes; and 2 amplicons, 1 at 529 bp and 1 at 379 bp in heterozygous mosquitoes.

### Genotyping *15xQUAS-CaMPARI2*

*15xQUAS-CaMPARI2* was genotyped using a multiprimer PCR assay with the forward primer: 5′-GTTTGACCAAATGCCGTTTC-3′ and 2 standard reverse primers: 5′-GTCGATAGGCGCGTAGTGTA-3′ and 5′-CACCACTTTTGAAGCGTTGA-3′. Each of the reverse primers was used at half the normal concentration. This resulted in a single 645-bp amplicon in homozygous mosquitoes; a single 874-bp amplicon in WT mosquitoes; and 2 amplicons, 1 at 645 bp and 1 at 874 bp in heterozygous mosquitoes.

### Transgenic stock maintenance and composite genotypes

*Gr1^QF2–3xP3^*, *Gr1^QF2-MF^*, *Ir8a^QF2–3xP3^*, and *Ir8a^QF2-MF^* driver lines were maintained as homozygous stocks. *orco^QF2–3xP3^* was maintained as a heterozygous stock by outcrossing to *LVPib12* each generation. *orco^QF2-MF^* was maintained as a heterozygous stock by outcrossing to either *LVPib12* or *QUAS-mCD8::GFP* each generation and screening for GFP fluorescence in olfactory tissues of the progeny. *15xQUAS-mCD8::GFP* and *15xQUAS-CaMPARI2* responder lines were maintained as homozygous stocks. The *exu-Cre* line was maintained as a heterozygous stock by outcrossing to *LVPib12* each generation. For long-term maintenance, we have determined that all driver and responder stocks except *orco^QF2–3xP3^* and *exu-Cre* are capable of being maintained as homozygous stocks. We do not observe any major fitness effects that preclude laboratory rearing of these stocks when maintained as indicated.

### Immunohistochemistry

Immunostaining of female *A. aegypti* brains was performed as previously described (Shankar and McMeniman 2020), with minor modifications. Briefly, severed mosquito heads were fixed in 4% paraformaldehyde (Milonig's buffer, pH 7.2) for 3 h after which brains were carefully dissociated from the head capsule, pigmented ommatidia, and air sacs. Dissected brains were then subjected to three 20-min washes at room temperature in PBST (0.1 M PBS with 0.25% Triton-X 100) and allowed to incubate overnight in a blocking solution consisting of 2% normal goat serum (NGS) and 4% Triton-X 100 in 0.1 M PBS at 4°C. Brains were then washed 3 times for 20 min each in PBST and incubated for 3 days at 4°C in a primary antibody solution containing mouse anti-BRP (DSHB, nc82-s, AB_2314866, 1:50 v/v) targeting the presynaptic active zone protein Bruchpilot (Hofbauer *et al*. 2009) and rabbit anti-GFP (Invitrogen, A-6455, 1:100 v/v) targeting mCD8::GFP. Brains were then washed 3 times for 20 min each in PBST and incubated for 3 days at 4°C in a secondary antibody solution consisting of goat antimouse Cy3 (Jackson ImmunoResearch, AB_2338680, 1:200 v/v) and goat antirabbit Alexa Fluor 488 (Invitrogen, A-11008, 1:200 v/v). All primary and secondary antibody dilutions were prepared in PBST with 2% v/v NGS. Brains were finally washed 3 times for 20 min each in PBST at room temperature and mounted in 20 µL of SlowFade Gold antifade mountant (Invitrogen, S36936) on glass slides with coverslip bridges (number 2–170 μm).

### Immunohistochemistry image acquisition settings

Brain immunostaining images were acquired on a single-point laser scanning Carl-Zeiss LSM 780 confocal microscope. To capture images of the entire adult brain, a 10× objective lens (0.3 NA, Plan-Apochromat) was used. Excitation of Cy3 signal was achieved with a 561-nm solid-state laser line at 0.05% laser power and GaAsP detector gain set to 825. A 488-nm laser line was used to excite Alexa Fluor 488 (20% laser power, detector gain at 825). We additionally acquired images with a 20× objective lens (0.8 NA, Plan-Apochromat) to perform 3D reconstructions of the antennal lobes. For these, the power of the 488-nm laser line was adjusted to 5%. For each antennal lobe, 60 z-slices with a z-step size of 1 μm and a 1,024 × 1,024-pixel resolution were acquired.

### Antennal lobe reconstructions

Three-dimensional morphological reconstructions of left antennal lobes were performed as previously described (Shankar and McMeniman 2020). Briefly, confocal images were imported into Amira (FEI Houston Inc.) and then segmented by highlighting all pixels across a z-stack occupied by individual glomeruli. The nc82 (Bruchpilot) channel was used for manual segmentation of individual glomeruli. The GFP channel was then used to identify *orco+*, *Ir8a+*, and *Gr1*+ glomeruli. Cross-referencing signals obtained from nc82 and GFP channels within and between samples in this dataset helped to clearly delineate glomerular boundaries. 3D and 2D antennal lobe models were generated by surface rendering. The number of GFP-labeled glomeruli in 3 replicate left antennal lobe reconstructions per genotype from *orco^QF2-MF^ > 30xQUAS-mCD8::GFP*, *Ir8a^QF2-MF^ > 30xQUAS-mCD8::GFP*, and *Gr1^QF2-MF^ > 30xQUAS-mCD8::GFP* females was counted. The total number of glomeruli per lobe was counted in 7 of these samples: *orco^QF2-MF^ > 30xQUAS-mCD8::GFP* (*n* = 3), *Ir8a^QF2-MF^ > 30xQUAS-mCD8::GFP* (*n* = 3), and *Gr1^QF2-MF^ > 30xQUAS-mCD8::GFP* (*n* = 1).

### Glomerular volume and frequency

Glomerular volumes were obtained from the left antennal lobe using the nc82 channel. To name glomeruli, we first identified landmark glomeruli in each antennal lobe sample using a systematic *A. aegypti* antennal lobe reference key (Shankar and McMeniman 2020), as this updated map better reflected glomerular organization in our dataset relative to a previous map (Ignell *et al*. 2005). Each antennal lobe glomerulus labeled with GFP was named based on its spatial position relative to these landmarks and flanking glomeruli. We classified glomeruli as spatially “invariant” or “variant” based on their frequency of identification. A threshold frequency of 80% or more across reconstructions was designated for the classification of spatially invariant glomeruli. Glomerular volume and frequency values were calculated from a pooled dataset consisting of the 7 reconstructions where we named all constitutive glomeruli in the same left antennal lobe samples used for glomerular counts.

### Confocal imaging of peripheral olfactory appendages

Live antenna, palp, and proboscis tissue were dissected in 0.1 M PBS and immediately mounted in SlowFade Gold antifade mountant (Invitrogen, S36936). Images were acquired on a Carl-Zeiss LSM 780 confocal microscope within 1 h of dissection. To excite the *GFP* signal, the 488-nm laser line was used at 5% laser power. An additional DIC channel was used to visualize gross morphology of the peripheral tissue. Images of the antennae were acquired with a 20× objective lens (0.8 NA, Plan-Apochromat), while images of the palp and labella of the proboscis were taken with a 40× (1.3 NA, Plan-Apochromat) oil immersion objective.

### Mosquito preparation for peripheral calcium imaging

Five- to 10-day-old female mosquitoes were transferred to plastic fly vials (Flystuff, 32–110) containing a cotton ball with distilled water for fasting (1 mosquito per vial). The vials were placed in a climate-controlled incubator (27°C, 80% relative humidity) for 20–24 h before testing. Mosquitoes were cold anesthetized for 3 min, and the wings and legs were removed. They were then placed on a 3D-printed holder that allowed for the mosquito to rest upside down, exposing the ventral side of the palps where the CO_2_ sensing neurons are found. The dorsal side of the palps was placed on double-sided tape (Scotch, 137DM-2) to fix them in position. The proboscis was taped down to reduce movement of the preparation.

### Mosquito preparation for antennal lobe recordings

Five- to 10-day-old female mosquitoes were fasted as described above. Females were briefly knocked out on ice, and legs and wings were removed. They were then situated within a small oval cut into tin foil that was just big enough so that the top of the head and thorax rested above the tin foil and the olfactory organs and rest of the body were below and free to move. UV glue (BONDIC CECOMINOD032561) was applied with a thin tungsten wire to secure the upper thorax and head to the tin foil. Next, calcium-free saline (150 mM NaCl, 3.4 mM KCl, 5 mM glucose, 1.8 mM NaHCO_3_, 1 mM MgCl_2_, 25 mM HEPES; pH 7.1; 0.22 µm filtered sterilized) was added to cover the top of the head and a 31-gauge needle (BD 328289) was used to cut a square through the cuticle from the base of the pedicels to the vertex. The cuticle was gently removed with sharp forceps, and calcium-free saline was then replaced with saline with calcium (same saline as above with 1.7 mM CaCl_2_) by removal with a Kimwipe and simultaneous addition of new saline with a pipette at which point imaging commenced.

### CO_2_ delivery for calcium imaging

5% CO_2_ (X02AI95C2000117, Airgas) was mixed with clean air (UZ300, Airgas) using mass flow controllers (MC-series 100, MC-series 500, Alicat Scientific) to obtain the desired concentration at a flow of 2 mL/s. The stimulus was controlled by a solenoid valve (ETO-3-12, Clippard) and valve driver (Automate Scientific, ValveLink 8.2) and brought into a carrier flow of humidified air flowing at 6 mL/s for a total flow of 8 mL/s. A compensatory clean air flow was on at 2 mL/s whenever the stimulus was off to ensure a constant flow of 8 mL/s.

### Calcium imaging system and analysis

Calcium signals were measured using an epifluorescence microscope (BX51W1, Olympus) and an LED light source (Lambda HPX-L5, Sutter Instrument) at 50× magnification for peripheral recordings and 20× for AL recordings. Recordings were carried out at 30 fps using a CMOS camera (Orca-Fusion C14440, Hamamatsu Photonics K.K.) controlled by micro-manager. The videos were analyzed using FIJI, and the data were analyzed using MATLAB R2018b (The MathWorks, Inc.).

### Live mosquito preparation for CaMPARI2 photoconversion

To prepare mosquitoes for CaMPARI2 photoconversion (Moeyaert *et al*. 2018), mosquitoes were cold anesthetized and tethered to an imaging chamber. To do this, the thorax of a female mosquito was first affixed to the ventral surface of a 35-mm petri dish lid (Eppendorf, 0030700112) using UV-curing adhesive (Bondic) immediately next to a 15-mm-diameter circular hole made in the lid center. Two additional drops of adhesive were applied to the ommatidia on the extremities of the mosquito head to prevent head movement. A small piece of clear tape (Duck EZ Start, Heavy Duty Packaging Tape) was then adhered over the center hole. The dorsal surface of the mosquito head was then gently affixed to the ventral adhesive tape surface covering the hole. An excised section of plastic coverslip (5 mm × 3 mm) was then affixed to the tape and used to shield the antennae from the adhesive tape surface and suspend these sensory appendages in the air.

The imaging chamber with head-fixed mosquito was then inverted, and a rectangular incision ∼400 µm × 200 µm was cut through the tape window where the dorsal head cuticle and ommatidia were affixed. The wide boundary of the incision was typically made immediately adjacent to the first antennal subsegment along the lateral–medial brain axis, while the short boundary of the incision extended along the dorsal–ventral brain axis. To create this window, segments of ommatidia and bridge cuticle between the left and right eyes were gently cut and excised using a surgical stab knife (Surgical Specialties Corporation, Sharpoint, Part # 1038016) to reveal the underlying antennal lobes. The exposed antennal lobes were then immediately immersed in an *A. aegypti* Ringer's solution (Beyenbach and Masia 2002) composed of 150 mM NaCl, 3.4 mM KCl, 5 mM glucose, 1.8 mM NaHCO_3_, 1 mM MgCl_2_, 25 mM HEPES, and 1.7 mM CaCl_2_; pH 7.1. Mosquitoes were allowed to recover for a period of 15 min from cold anesthesia and surgery in a humidified chamber at room temperature prior to imaging.

### CaMPARI2 photoconversion

For CaMPARI2 photoconversion, the tethered preparation was placed under a 20× water dipping objective (Olympus XLUMPLFLN20XW, 1.0 NA), ensuring that the antennal lobes expressing basal green CaMPARI2 signal were in focus. Each preparation was exposed to a combined photoconversion-odor stimulation regime consisting of repetitive duty cycles of four 500-ms pulses of 405-nm light from an LED driver (Thorlabs, DC4104, 1,000 mA current setting) synchronized with a 1-s odorant pulse as previously outlined (Fosque *et al*. 2015), for 75 cycles with a total protocol duration of ∼41 min.

### Odorant delivery for CaMPARI2 photoconversion

A 1-mL/s stream of 5% CO_2_ was diluted 1:5 into the carrier airstream for a final concentration at the specimen of 1% CO_2_ and was then piped via Teflon tubing into a carrier airstream of humidified synthetic air that was directed at the olfactory appendages of the mosquito using a plastic pipette. During CaMPARI2 photoconversion assays, the tethered mosquito preparation always received a constant amount of airflow (5 mL/s) during odor onset/offset from the stimulus pipette. This was achieved via solenoid valves simultaneously switching or combining humidified synthetic air, 5% CO_2_ (Airgas).

### CaMPARI2 sample processing

Following photoconversion, the mosquito was gently untethered from the imaging chamber and the head severed and fixed in Milonig's buffer for 20 min. The brain was then dissected out in calcium-free Ringer's solution composed of 150 mM NaCl, 3.4 mM KCl, 5 mM glucose, 1.8 mM NaHCO_3_, 1 mM MgCl_2_, 25 mM HEPES, and 10 mM EGTA. To stain glomerular boundaries, we incubated each brain in Alexa Fluor 647 Phalloidin (Invitrogen, A22287) prepared in calcium-free Ringer's solution (1:40 v/v dilution) for 30 min. To prepare Alexa Fluor 647 phalloidin for use in imaging, first, a 400× DMSO stock solution was prepared according to the manufacturer's instructions by dissolving the fluorophore in 150 µL of DMSO. One microliter of this DMSO stock was diluted in 399 µL calcium-free Ringer's solution to yield a 1× stock. This 1× stock was then further diluted 1:40 in calcium-free Ringer's solution for staining. Brains were transferred directly from this solution into 20 µL of SlowFade Gold antifade mountant (Invitrogen, S36936) on glass slides with coverslip bridges (number 2–170 μm) for CaMPARI2 and phalloidin imaging.

### CaMPARI2 image acquisition settings

Antennal lobes from CaMPARI2 photoconversion assays were imaged with a 63× (1.4 NA) oil immersion objective on a Zeiss 880, Airyscan FAST super-resolution single-point scanning microscope. Excitation of red CaMPARI2 signal was achieved with a 561-nm solid-state laser line at 14% laser power. Green CaMPARI2 was excited with a 488-nm argon laser line at 10% laser power. To visualize glomerular boundaries, a 633-nm diode laser was used to excite the Alexa-647 phalloidin fluorophore at 40% laser power. Master detector gain was set to a value of 800. We captured 0.987 μm z-slices of 1,572×1,572-pixel resolution in the FAST mode. Raw images were further processed by applying the Airyscan method with “auto” processing strength.

### CaMPARI2 image analysis

Image analysis was carried out in Fiji (http://imagej.net/Fiji). We first applied a median filter (radius = 2 pixels) to remove noise and then a rolling ball subtraction (rolling ball radius = 80 pixels) to correct for nonuniformity of background intensities. ROIs were defined by manually segmenting the MD1 glomerulus using the freehand selection tool. The integrated density (mean gray value × area) for all z-slices of the ROI, which included all representative slices of a target glomerulus, was calculated in the green (488 nm) and red (560 nm) imaging channels. The final measure of photoconversion, the red-to-green ratio photoconversion (*R*/*G*), was calculated as:


$$\begin{array}{rl} R/G & =\mathrm{Average}\;\mathrm{integrated}\;\mathrm{density}\;\mathrm{of}\;\mathrm{RO}I^{\left(\mathrm{RED}\right)}\\ & \;/\mathrm{Average}\;\mathrm{integrated}\;\mathrm{density}\;\mathrm{of}\;\mathrm{RO}I^{\left(\mathrm{GREEN}\right)} \end{array}$$

## Statistical analysis

We tested normality of raw data with a Kolmogorov–Smirnov test. To test for the statistical significance of differences observed in different experiments, dependent on context we used Wilcoxon rank-sum, Mann–Whitney U and paired t-tests, and ANOVA with post hoc comparisons. Statistical analyses were performed in MATLAB R2018b (The MathWorks, Inc.) and GraphPad Prism Software version 8.4.0. Details of statistical methods are reported in the figure legends.

## Results

### Targeted genetic access to *A. aegypti* OSN populations using CRISPR–Cas9 HDR donors that report the nature of transgene integration

CRISPR–Cas9-mediated *T2A* in-frame fusions (Diao and White 2012) are a broadly used genetic strategy to capture endogenous expression patterns of target genes of interest in varied organisms, including *A. aegypti*, where this technology is routinely applied to map the expression patterns of hygrosensors, thermoreceptors, chemoreceptors, and other neural genes (Matthews *et al*. 2019; Jové *et al*. 2020; Zhao *et al*. 2021, 2022; Herre *et al*. 2022; Laursen *et al*. 2023). Of note, all existing *T2A-QF2* integrations reported to date in *A. aegypti* have employed *3xP3* fluorescent transgenesis markers (Berghammer *et al*. 1999) to identify transformants. With current designs employed in *A. aegypti*, these *3xP3* transgenesis markers cannot be easily removed post-integration due to a lack of specific sequence motifs flanking these cassettes to facilitate their excision. Furthermore, current donor construct designs used for CRISPR–Cas9-mediated HDR at target loci in *A. aegypti* do not report the nature of cassette integration.

Specifically, 2 common integration outcomes may occur in the presence of a double-stranded break and circular homologous donor plasmid during CRISPR–Cas9-mediated HDR across various species. These include canonical HDR repair events, as well as noncanonical insertions in which the intended insertion is duplicated at the target site, with the duplicate copies separated by intervening plasmid sequence. This latter phenomenon has been observed in different organisms including *Drosophila* (Bier *et al*. 2018; Zirin *et al*. 2022; Loehlin *et al*. 2023). Within the context of *T2A* in-frame fusions, canonical HDR repair events result in precise integration of a single copy of the *T2A-QF2* donor cassette into the target site. On the other hand, noncanonical duplications, while also yielding a precise integration of *T2A-QF2* into the target site as intended, may complicate genotyping of lines post-establishment as the plasmid backbone and a duplicate copy of the donor cassette are also integrated.

**Fig. 1.**
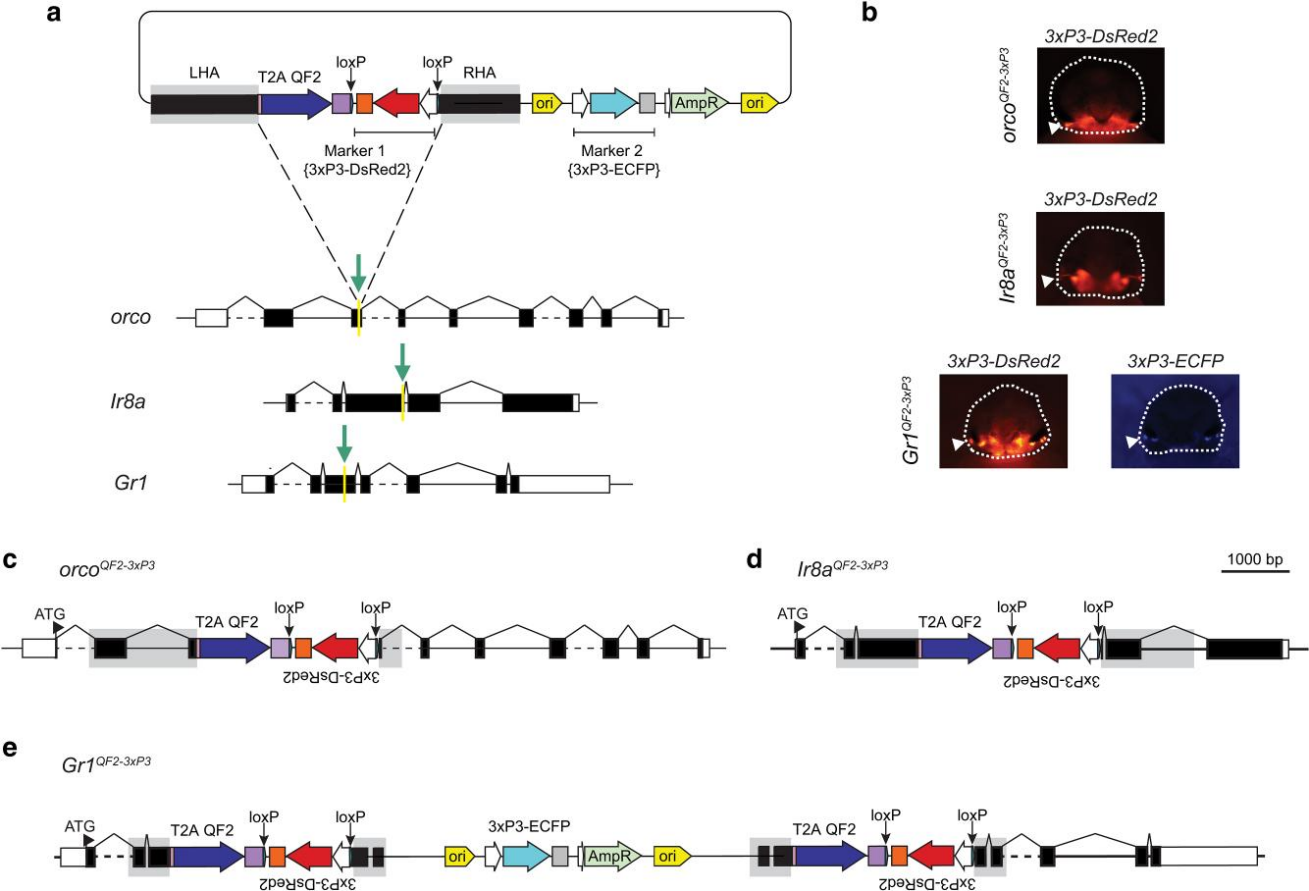
A dual transgenesis marker system reports canonical and noncanonical HDR repair events during CRISPR–Cas9-facilitated *T2A-QF2* in-frame fusions in *A. aegypti* chemoreceptor genes. a) Schematic of circular donor plasmid used for CRISPR–Cas9-mediated HDR to generate *T2A-QF2* in-frame fusion driver lines for the *A. aegypti orco*, *Ir8a*, and *Gr1* genes. The targeting construct contains a *3xP3-DsRed2* transgene (transgenesis marker 1) flanked by loxP sites in the intended integration cassette and an additional *3xP3-ECFP* transgene (transgenesis marker 2) positioned in the vector backbone. LHA, left homology arm; RHA, right homology arm; gRNA target site for each gene indicated by arrows. b) Marker expression in the head of fourth-instar larvae from *orco^QF2–3xP3^*, *Ir8a^QF2–3xP3^*, and *Gr1^QF2–3xP3^* driver lines. Heads outlined with dashed lines, arrows indicate marker expression in larval eyes c, d) Schematics of the canonical HDR integration events that generated the *orco^QF2–3xP3^* and *Ir8a^QF2–3xP3^ T2A-QF2* driver lines which are marked by only a single *3xP3-DsRed2* transgene flanked by loxP sites. e) Schematic of the noncanonical integration event that generated the *Gr1^QF2–3xP3^* driver line, marked by 2 *3xP3-DsRed2* transgenes flanked by loxP sites, separated by intervening vector sequence with a *3xP3-ECFP* transgene. The donor cassette is duplicated. Homology arms are shaded, and the positioning of loxP sites is indicated with arrows in a) and c—e).

We therefore updated the design of our circular homologous recombination construct used for *T2A-QF2* in-frame fusions in *A. aegypti* to include a *T2A-QF2* cassette with a *3xP3-DsRed2* eye marker flanked by loxP sites (floxed), as well as a second nonfloxed *3xP3-ECFP* eye marker in the vector backbone (Fig. 1a). This construct design facilitates the rapid identification of transgene integrations generated by canonical HDR (i.e. those with DsRed2 positive eyes only) and those that also possibly incorporate plasmid backbone indicative of a multiplexed integration event of the donor cassette (i.e. those with DsRed2- and ECFP-positive eyes). In this donor construct design, loxP sites flanking the *3xP3-DsRed2* eye marker also enable marker removal from canonical integration events using Cre-loxP excision, which is highly efficient in *A. aegypti* (Häcker *et al*. 2017). Similarly, duplication of the floxed *3xP3-DsRed2* donor cassette and insertion of the intervening plasmid backbone sequence, including the *3xP3-ECFP* marker, from multiplexed noncanonical recombination events can be resolved to yield an integration reflective of canonical HDR using this excision method.

To generate transgenic *A. aegypti* chemoreceptor-*QF2* driver lines using this updated targeting strategy, we first designed gRNAs and circular HDR donor constructs to integrate *T2A-QF2* in-frame into the coding exons of 2 olfactory co-receptor genes: *orco* and *Ir8a* which have been targeted previously using alternative construct designs (Herre *et al*. 2022; Zhao *et al*. 2022), as well as the CO_2_ receptor complex gene *Gr1* (McMeniman *et al*. 2014) which has not been targeted before (Fig. 1a). Using this strategy, *QF2* was precisely integrated in-frame into Exon 3 of each target gene, placing the expression of this transcription factor under control of the endogenous regulatory elements for each locus. Using this approach, we successfully recovered precise *T2A* in-frame fusion integrations in *orco* and *Ir8a* via canonical HDR as indicated by the presence of a *3xP3-DsRed2* marker only in these lines (Fig. 1b–d). We also obtained a precise T2A in-frame fusion integration event in *Gr1* that included 2 copies of the *T2A-QF2* donor cassette and intervening plasmid backbone as indicated by the presence of both *3xP3-DsRed2* and *3xP3-ECFP* markers in transformants (Fig. 1b and e). The orientation of this latter insertion in the *A. aegypti* genome was verified using long-read Nanopore sequencing, and we further confirmed that this sequence orientation was not found in the original donor plasmid using the same method and therefore was the result of noncanonical HDR (Fig. 1e).

Of note during isolation of the above *T2A-QF2* lines, in the G_1_ generation post-microinjection we observed only noncanonical HDR events for *Gr1*, a mixture of canonical and noncanonical HDR events for *Ir8a*, and a single canonical HDR event for *orco* (Supplementary Table 6 in Supplementary File 1). These differing HDR repair outcomes were also observed across different formats of Cas9 delivery and microinjection suppliers (Supplementary Table 6 in Supplementary File 1). We proceeded to characterize a single noncanonical HDR event carrying both *3xP3-DsRed2* and *3xP3-ECFP* transgenesis markers for the *Gr1* integration as that was the only option. Given their availability, single representative lines were selected for downstream characterization for *Ir8a* and *orco* integrations which each solely carried a *3xP3-DsRed2* transgenesis marker.

**Fig. 2.**
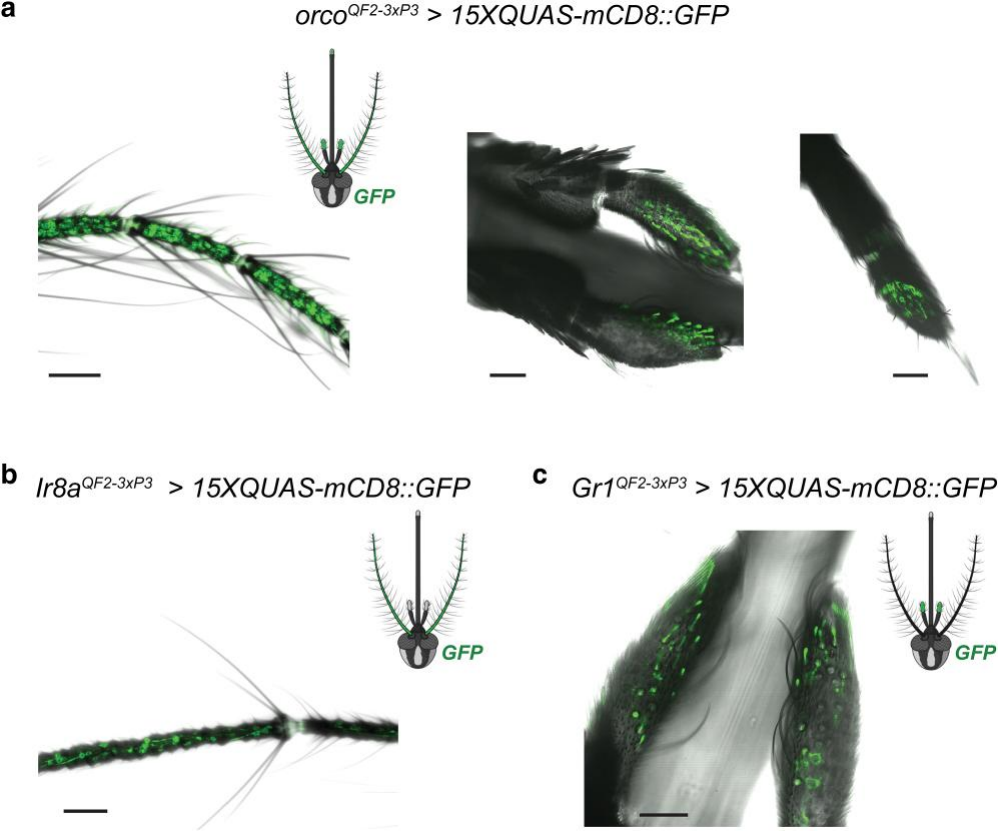
*T2A-QF2* in-frame fusions facilitate visualization of *orco*+, *Ir8a*+ and *Gr1*+ OSNs on the olfactory appendages of *A. aegypti*. OSNs were labeled with membrane-tethered GFP (mCD8::GFP) in the: a) antennae (left), maxillary palps (middle), and labella of the proboscis (right) of *orco^QF2–3xP3^ > 15xQUAS-mCD8::GFP* females. b) Antennae of *Ir8a^QF2–3xP3^ > 15xQUAS-mCD8::GFP* females, and c) maxillary palps of *Gr1^QF2–3xP3^ > 15xQUAS-mCD8::GFP* females. A schematic of the mosquito head with organs exhibiting observable GFP fluorescence for each line is shown. Scale bars: 50 µm.

Each of these *3xP3*-marked chemoreceptor-*QF2* driver lines (*orco^QF2–3xP3^*, *Ir8a^QF2–3xP3^*, and *Gr1^QF2–3xP3^*) was crossed with a *15xQUAS-mCD8::GFP* responder strain that we generated via Mos1 mariner transposition (Coates *et al*. 1998; Supplementary Table 7 in Supplementary File 1). In contrast to the only other currently available *QUAS-mCD8::GFP* responder strain for this species, which includes a *SV40* terminator for the *mCD8::GFP* gene (Matthews *et al*. 2019; Herre *et al*. 2022), we flanked this fluorescent reporter with both *Syn21* and *p10* 3′UTR sequences, shown to act as translational enhancers in *Drosophila* (Pfeiffer *et al*. 2012) to generate a line that robustly labels neuronal membrane and processes in this mosquito. Olfactory tissues from the F_1_ progeny of these crosses were then surveyed for the presence of membrane-tethered GFP in OSNs. Confocal analyses of adult peripheral sensory appendages revealed strong GFP labeling of *orco* + OSN dendrites and cell bodies on the antenna, maxillary palp and labella of the proboscis of *orco^QF2–3xP3^* > *15xQUAS-mCD8::GFP* females (Fig. 2a). Strong labeling of *Ir8a* + OSNs within the antennal flagella (Fig. 2b) of *Ir8a^QF2–3xP3^* > *15xQUAS-mCD8::GFP* and that of *Gr1*+ OSNs in maxillary palp tissue (Fig. 2c) of *Gr1^QF2–3xP3^* > *15xQUAS-mCD8::GFP* females were also detected.

Expression patterns from these *T2A-QF2* knock-ins were consistent with a previous *A. aegypti LVPib12* strain neurotranscriptome analysis using bulk RNAseq (Matthews *et al*. 2016) that revealed broad *orco* expression across adult olfactory tissues, along with strong, tissue-specific expression of *Ir8a* and *Gr1* in antennal and maxillary palp tissue, respectively. The peripheral GFP labeling patterns that we observed for our *orco^QF2–3xP3^* and *Ir8a^QF2–3xP3^* lines developed here were also consistent with those observed for other *T2A-QF2* in-frame fusions recently generated in the last coding exon of these genes (Herre *et al*. 2022). The pattern of GFP labeling that we observed for our *Gr1^QF2–3xP3^* driver line also mirrored that of another *T2A* in-frame fusion in the last coding exon of *Gr3* (Herre *et al*. 2022), with both lines labeling OSNs localized to capitate peg sensilla in the *A. aegypti* maxillary palps.

The complementary transgenic lines developed here thus expand the toolkit to genetically access and label target *A. aegypti* OSN populations. Using these new *T2A-QF2* driver lines and our *15xQUAS-mCD8::GFP* responder line equipped with translational enhancers, we conclude that at the resolution of transgenic labeling and confocal imaging performed here, *orco+* neurons clearly innervate all 3 olfactory sensory appendages in *A. aegypti*, whereas *Ir8a+* and *Gr1+* OSNs strongly innervate the antennae and maxillary palps, respectively.

### A mutant *A. aegypti* strain with reduced cuticular pigmentation enables sensitive transcuticular imaging of OSN activity

To initially validate the applicability of these *T2A-QF2* in-frame fusions for functional imaging of neural activity in *A. aegypti*, we first performed peripheral calcium imaging from CO_2_ neurons on the ventral surface of the maxillary palps. The maxillary palps are the anatomical site of CO_2_ detection in this species (Kellogg 1970; Grant *et al*. 1995), where the dendrites and underlying cell bodies of capitate peg A (cpA) neurons that co-express the CO_2_ receptor complex subunits Gr1, Gr2, and Gr3 are localized (Jones *et al*. 2007; Lu *et al*. 2007).

To do this, we crossed our *Gr1^QF2–3xP3^* driver with a *15xQUAS-Syn21-GCaMP6s-p10* responder strain (*15xQUAS-GCaMP6s*) that we engineered via Mos1 mariner transposition (Supplementary Table 7 in Supplementary File 1), and imaged maxillary palp tissue in F_1_ progeny carrying both transgenes. Although peripheral calcium imaging has been successfully used to study stimulus-evoked activity in *A. coluzzii* and *A. gambiae* antenna and maxillary palps (Afify *et al*. 2019; Laursen *et al*. 2023; Raji *et al*. 2023; Giraldo *et al*. 2024) and responses to tastants in the dissected stylet of *A. aegypti* (Jové *et al*. 2020), we observed that the darker pigmentation of the *A. aegypti* cuticle posed a major challenge toward measuring changes in GCaMP6s fluorescence in response to stimulation with CO_2_. In particular, we hypothesized that this cuticular barrier optically occluded and dampened odor-evoked changes in fluorescence.

**Fig. 3.**
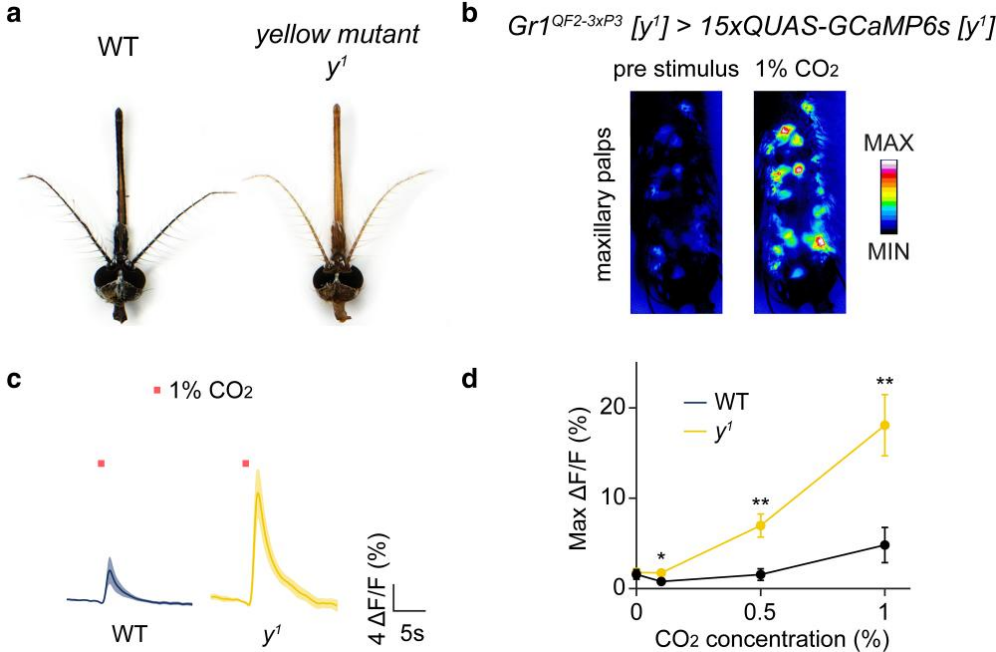
*Aedes aegypti* cuticular pigmentation mutant enables peripheral calcium imaging of CO_2_-evoked activity from *Gr1*+ OSNs on the maxillary palps with enhanced sensitivity. a) Female *A. aegypti* of WT (left panel) and *yellow* mutant [*y^1^*] (right panel) backgrounds. b) GCaMP6s fluorescence in maxillary palp neurons before (left panel) and during 1% CO_2_ stimulation (right panel). c) GCaMP6s traces of CO_2_-sensitive neurons in the maxillary palps after a 1% CO_2_ pulse for 1 s (bar) from WT (*Gr1^QF2–3xP3^ > QUAS-GCaMP6s*) and *yellow* mutant (*Gr1^QF2–3xP3^ [y^1^] > QUAS-GCaMP6s [y^1^]*) females. The solid line represents the mean and the shaded area the standard error of the mean (*n* = 10 WT, *n* = 11 *yellow*). d) Maximum change in fluorescence to increasing concentrations of 1-s CO_2_ pulses in WT and *y^1^* females. The dots represent the mean, and the error bars the standard error of the mean (*n* = 10 WT, *n* = 11 *y^1^*). Wilcoxon rank-sum test, *P < 0.05, **P < 0.01.

As cuticle covering all sensory appendages of WT *A. aegypti* including the maxillary palps is highly melanized, we sought to lighten cuticular pigmentation by introgressing both our *Gr1^QF2–3xP3^* and *15xQUAS-GCaMP6s* transgenes into a *yellow* mutant background (Li *et al*. 2017). This was performed as the *yellow* gene is important for cuticular melanization in various insect species (Biessmann 1985; Wittkopp *et al*. 2002; Berni *et al*. 2022; Gong *et al*. 2024), and mutants of this gene in *A. aegypti* (Li *et al*. 2017) have lighter cuticle (Fig. 3a). We then compared odor-evoked changes in GCaMP6s fluorescence by stimulating *Gr1^QF2–3xP3^ > 15xQUAS-GCaMP6s* female mosquitoes in both *yellow* mutant (*y^1^*) and WT genetic backgrounds in response to stimulation with various concentrations of CO_2_.

In response to stimulation with CO_2_, we determined that clear odor-evoked changes in GCaMP6s florescence intensity could be observed from *Gr1+* OSNs in WT and *yellow* mutants (Fig. 3b). Strikingly, maximum odor-evoked changes in fluorescence from these cells were much higher in *yellow* mutants relative to WT (Fig. 3c). We next quantified maximum changes in GCaMP6s fluorescence intensity in response to stimulation with a range in CO_2_ concentrations reflective of those found in diluted human breath (0, 0.1, 0.5, and 1% CO_2_). We found that GCaMP6s responses were significantly elevated from *Gr1+* OSNs in *yellow* mutants (Fig. 3d). In contrast, WT mosquitoes failed to exhibit major increases in GCaMP6s fluorescence across these concentrations (Fig. 3d). Notably, *yellow* mutant mosquitoes exhibited a significant, 2–4-fold increase in the maximum change in fluorescence above baseline (Δ*F*/*F*_0_ values) relative to WT for each CO_2_ concentration tested.

Introgression of *QF2/QUAS* transgenes into the *yellow* mutant *A. aegypti* strain thus enhances the sensitivity of transcuticular imaging of CO_2_-evoked neural activity in this mosquito species over a range of gas concentrations.

### Excision of *3xP3* fluorescent markers resolves spurious labeling in the central mosquito brain

**Fig. 4.**
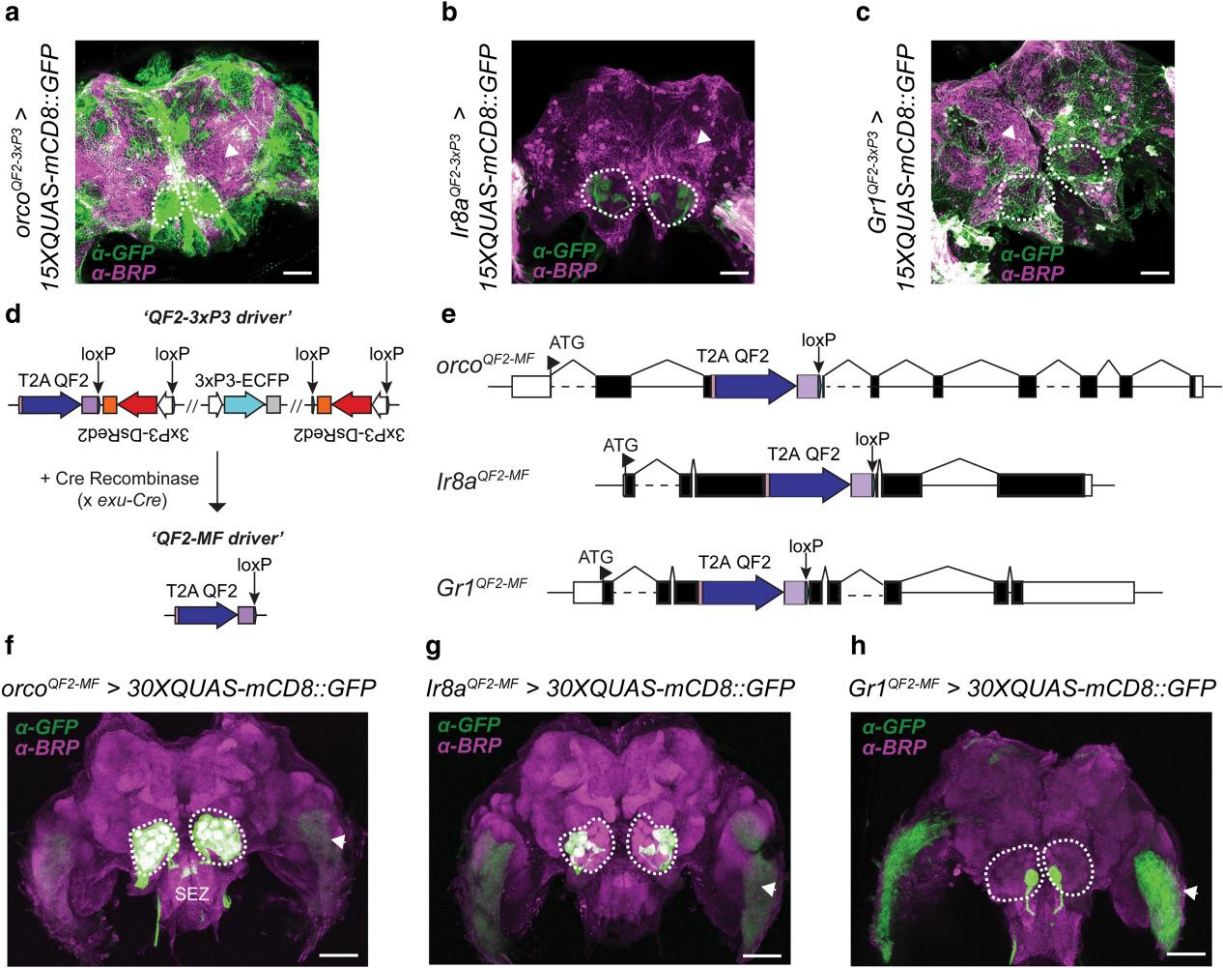
Cre-loxP-mediated excision of *3xP3* marker cassettes from chemoreceptor *T2A-QF2* driver lines facilitates clear visualization of OSN axonal projections into the *A. aegypti* antennal lobe. Spurious fluorescent labeling resulting from immunohistochemical analysis of adult female brains from a) *orco^QF2–3xP3^ > 15xQUAS-mCD8::GFP*; b) *Ir8a^QF2–3xP3^ > 15xQUAS-mCD8::GFP*; and c) *Gr1^QF2–3xP3^ > 15xQUAS-mCD8::GFP* genotypes. Anti-BRP (magenta), anti-GFP (green). Arrows indicate putative glial cells from background *3xP3-DsRed2* marker expression outside of the optic lobes in the central brain. d) Schematic of crossing scheme to facilitate Cre-loxP-mediated excision of *3xP3* marker cassettes from chemoreceptor *T2A-QF2^3xP3^* driver lines with canonical and noncanonical HDR integrations to yield marker-free *T2A-QF2^MF^* driver lines for central brain imaging. e) Schematic of resulting *orco^QF2-MF^*, *Ir8a^QF2-MF^*, and *Gr1^QF2-MF^* driver integrations. Positioning of loxP sites indicated by arrows for a–e). f, g) Maximum intensity projections for f) *orco^QF2-MF^ > 30xQUAS-mCD8::GFP* and g) *Ir8a^QF2-MF^ > 30xQUAS-mCD8::GFP* genotypes to visualize *orco*+ and *Ir8a*+ projections. h) Single posterior z-slice for the *Gr1^QF2-MF^ > 30xQUAS-mCD8::GFP* genotype to visualize *Gr1*+ projections is shown. Arrows indicate the expression of the *3xP3-ECFP* transgenesis marker for the *QUAS-mCD8::GFP* responder transgene in the outer optic lobes. Antennal lobes are encircled in white in a–c) and f–h). Confocal imaging settings were identical for all brains. SEZ, subesophageal zone. Scale bars: 50 µm. Images were taken at 20× magnification in a–c) and 10× magnification in f–h).

We next evaluated central projection patterns in these *3xP3*-marked chemoreceptor-*QF2* driver lines when crossed to our *15xQUAS-mCD8::GFP* responder described above. We expected that mCD8::GFP labeling would be restricted to axonal projections from each OSN class. Strikingly, immunohistochemical analysis of *orco^QF2–3xP3^ > 15xQUAS-mCD8::GFP*, *Ir8a^QF2–3xP3^ > 15xQUAS-mCD8::GFP*, and *Gr1^QF2–3xP3^ > 15xQUAS-mCD8::GFP* adult female brains revealed spurious red and green fluorescence throughout the central brain (Fig. 4a–c). This was broadly evident in multiple cell types across the brain, particularly in *orco^QF2–3xP3^ > 15xQUAS-mCD8::GFP* and *Gr1^QF2–3xP3^ > 15xQUAS-mCD8::GFP* genotypes, suggesting potential background interference in the expected pattern of *QF2/QUAS*-based transactivation. As all of our *T2A-QF2* insertions included downstream fluorescent marker cassettes containing the *3xP3* synthetic promoter (Berghammer *et al*. 1999), which is a multimerized binding site for the paired-box transcription factor *Pax6* involved in glial and neuronal development (Quiring *et al*. 1994; Suzuki *et al*. 2016), we suspected such aberrant expression patterns may be due to promiscuous *3xP3* enhancer activity operating at these genomic loci.

To abrogate this effect, we next excised the floxed *3xP3* fluorescent marker cassettes from these initial *T2A-QF2^3xP3^* strains by crossing these genotypes to a germline *Cre* recombinase strain (*exu-Cre*) that we engineered (Supplementary Table 7 in Supplementary File 1) to express Cre recombinase under the control of maternal germline promoter *exuperantia* (Li *et al*. 2017). We crossed males of each *T2A-QF2^3xP3^* driver line (*Ir8a^QF2–3xP3^*, *orco^QF2–3xP3^*, *Gr1^QF2–3xP3^*) to *exu-Cre* females and screened F_1_ progeny for loss of the *3xP3* fluorescent eye markers (Fig. 4d). In the case of the *Gr1^QF2–3xP3^* line, the duplicated *3xP3-DsRed2* marker due to the noncanonical HDR event was incompletely removed in F_1_ progeny, so progeny still containing visible DsRed2 or ECFP markers were mated to their *exu-Cre* + siblings to ensure complete excision of all markers. Using this approach, we successfully generated marker-free driver strains (*orco^QF2-MF^*, *Ir8a^QF2-MF^* and *Gr1^QF2-MF^*) which were devoid of all *3xP3* fluorescent markers (Supplementary Fig. 1 in Supplementary File 1), with only *T2A-QF2* and a downstream loxP site remaining after excision at each locus (Fig. 4e).

We next generated a series of *A. aegypti* genotypes for imaging central brain neuroanatomy: *orco^QF2-MF^ > 30xQUAS-mCD8::GFP*, *Ir8a^QF2-MF^ > 30xQUAS-mCD8::GFP*, and *Gr1^QF2-MF^ > 30xQUAS-mCD8::GFP*. Each of these *A. aegypti* strains had 1 copy of each marker-free *QF2* driver and 2 copies of the *15xQUAS-Syn21-mCD8::GFP-p10* transgene (i.e. for a cumulative dosage of *30xQUAS* to express membrane-tethered GFP to label OSNs). Using immunohistochemistry analyses with a primary antibody directed against the presynaptic protein Bruchpilot (BRP) (Hofbauer *et al*. 2009) to demarcate glomerular boundaries of neuropils in the antennal lobe, and an anti-GFP antibody to amplify mCD8::GFP signal, we clearly labeled OSN axonal projections in the central *A. aegypti* brain without the spurious labeling seen with the *3xP3*-marked driver lines. Of note, we determined *orco*+ neurons innervate a majority of antennal lobe glomeruli (Fig. 4f) and send axonal projections to the subesophageal zone (SEZ) (Supplementary Fig. 3 in Supplementary File 1). In contrast, *Ir8a*+ neurons project to several glomeruli in the posterior–lateral region of the antennal lobe (Fig. 4g), while *Gr1*+ neurons innervate a single glomerulus positioned deep in the posterior of the *A. aegypti* antennal lobe (Fig. 4h). These GFP labeling patterns were specific to each marker-free driver line, and we did not observe any apparent background fluorescence derived from OSNs innervating the peripheral sensory organs and antennal lobes of our *30xQUAS-mCD8::GFP* only control strain (Supplementary Fig. 2a–d in Supplementary File 1).

We conclude that *3xP3* markers that are commonly used for *A. aegypti* transgenesis have the potential to induce spurious background expression in the central brain and that this can be resolved by their precise excision. We demonstrate that application of this method facilitates cell-type-specific labeling of OSN projections in the *A. aegypti* central brain with clarity.

### An in vitro receptor-to-glomerulus map as the basis for improved *A. aegypti* antennal lobe annotation

We next performed confocal imaging and complete 3D morphological reconstructions of antennal lobes on replicate brain samples with labeled projections from *orco*+, *Ir8a*+, and *Gr1*+ OSNs using our marker-free *T2A-QF2 A. aegypti* genotypes. Across this dataset, we defined ∼79 total glomeruli (79 ± 3, mean ± SEM) in each reconstructed antennal lobe (Supplementary Fig. 4a in Supplementary File 1). This count was consistent with our previous estimate of ∼80 total glomeruli constituting the female *A. aegypti* antennal lobe based on reconstructions with synaptic staining alone (Shankar and McMeniman 2020). On average, we counted ∼63 *orco*+ glomeruli (63 ± 1, mean ± SEM), 15 *Ir8a*+ glomeruli, and 1 *Gr1*+ glomerulus in the reconstructed antennal lobes from each genotype (Supplementary Fig. 4b in Supplementary File 1).

Using a systematic reference key for *A. aegypti* antennal lobe nomenclature (Shankar and McMeniman 2020), we previously determined that 63 out of 80 total glomeruli (∼79%) could be found in stereotypical spatial positions in this mosquito species based on synaptic staining with anti-BRP antibody (nc82) alone. We determined here with transgenic labeling that cross-referencing BRP and GFP signals markedly improved our ability to define glomerular boundaries and discern the spatial arrangement of glomeruli within the antennal lobe in vitro. Indeed, we determined that 74 out of the 79 total glomeruli (∼94%) that we annotated could now be assigned to spatially conserved positions across reconstructions (Supplementary Fig. 4c and d in Supplementary File 1). A small proportion of glomeruli in each antennal lobe could not be reliably assigned to conserved spatial positions using our reference key and were classified as variant glomeruli (Supplementary Fig. 4c and e in Supplementary File 1).

**Fig. 5.**
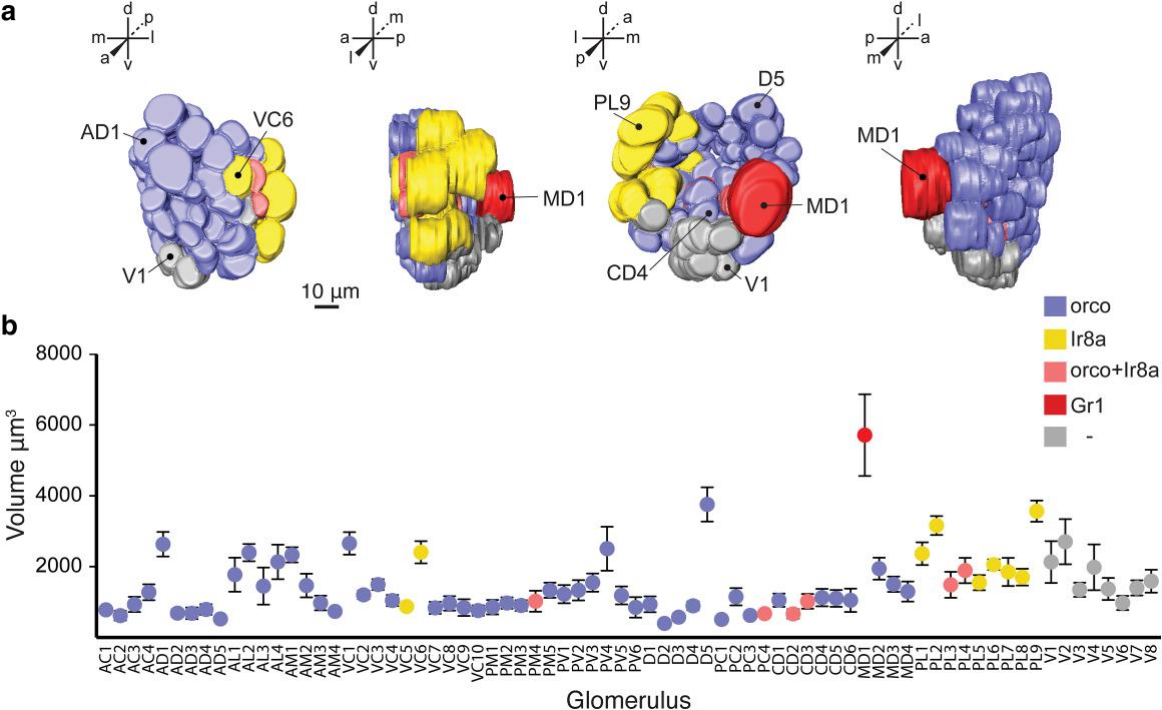
*Gr1*+ OSNs innervate the largest glomerulus in the *A. aegypti* antennal lobe. a) 3D reconstructed model of the left antennal lobe of a female *A. aegypti* mosquito as seen from the anterior, lateral, posterior and medial perspectives. Template genotype for model: *orco^QF2-MF^ > 30xQUAS-mCD8::GFP*. Landmark glomeruli are indicated. b) Glomerular volumes from the female left antennal lobe. Mean volumes ± SEM from spatially invariant glomeruli are plotted, *n* = 7 brains. Volumes varied significantly (1-way ANOVA, *P* < 0.0001). Tukey's multiple comparison test, *P* < 0.05 for all comparisons to the CO_2_ receptor glomerulus MD1.

We also determined that the region of the antennal lobe previously classified as the Johnston's Organ Center (JOC) (Ignell *et al*. 2005) appears to comprise of multiple discrete glomeruli in the postero-ventral and ventro-central groups of our updated atlas (Shankar and McMeniman 2020) that are innervated predominantly by *orco+* neurons and to a lesser extent *Ir8a+* neurons (Supplementary Fig. 4c in Supplementary File 1). Furthermore, volumetric analysis of glomeruli revealed that the *Gr1*+ glomerulus, denoted in both our atlas and prior atlases as MD1 (Ignell *et al*. 2005; Shankar and McMeniman 2020) or Glomerulus 1 (Herre *et al*. 2022), is the largest glomerulus in the antennal lobe (Fig. 5). Using spatial mapping, we also estimate a subset of 6 out of these 74 spatially invariant glomeruli are putatively both *orco*+ and *Ir8a*+ (Fig. 5 and Supplementary Fig. 4c and d in Supplementary File 1), consistent with recent observations of co-expression of these 2 genes in some OSN types (Herre *et al*. 2022).

The refined receptor-to-glomerulus map presented here thus reveals OSN populations expressing these 3 divergent chemoreceptors project centrally to defined regions of the *A. aegypti* antennal lobe.

### The largest glomerulus in the *A. aegypti* antennal lobe detects carbon dioxide

**Fig. 6.**
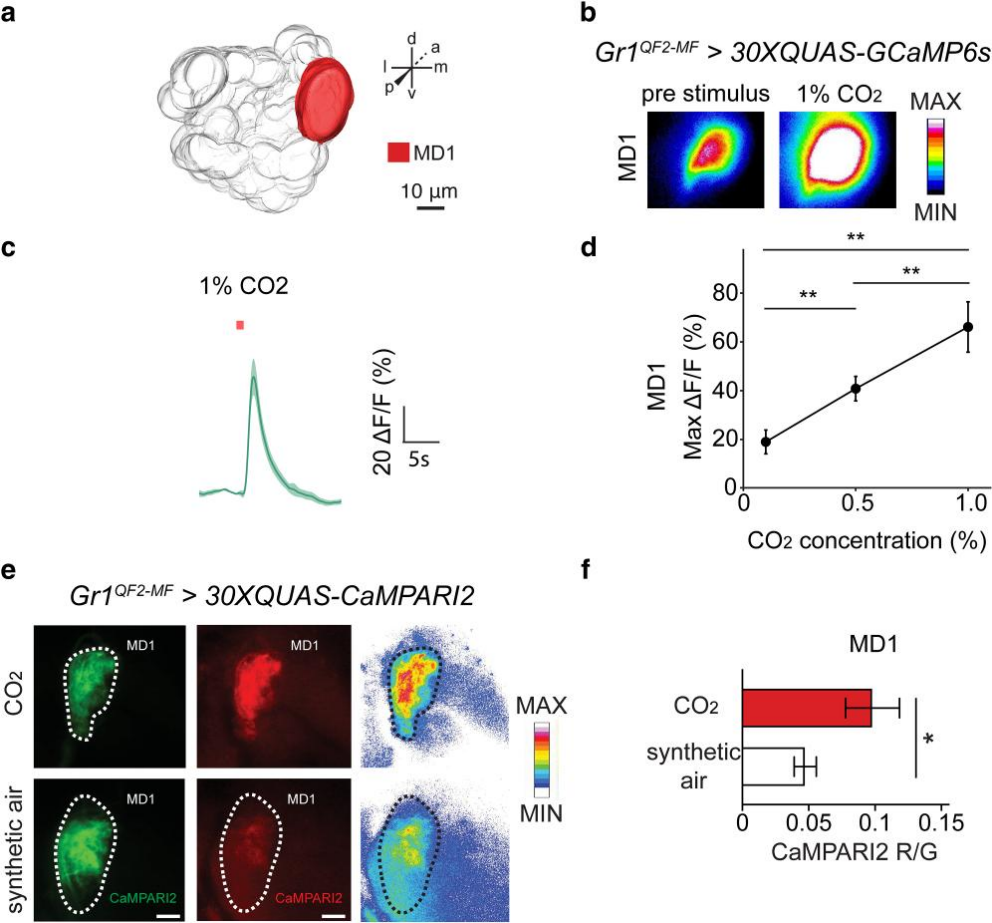
*Gr1*+ OSNs that project to the antennal lobe respond to CO_2_. a) 3D reconstruction of the *A. aegypti* left antennal lobe. *Gr1*+ neurons project to a large posteriorly positioned glomerulus called MD1. b) GCaMP6s fluorescence in the right MD1 glomerulus before (left panel) and during 1% CO_2_ stimulation (right panel). c) GCaMP6s traces after a 1% CO_2_ pulse for 1 s (red bar) in the right MD1 glomerulus (*Gr1^QF2-MF^ > QUAS-GCaMP6s*). The solid line represents the mean and the shaded area the standard error of the mean (*n* = 9). d) Maximum change in fluorescence in the right MD1 glomerulus in response to 1-s pulses of increasing CO_2_ concentration. The dots represent the mean, and the error bars the standard error of the mean (*n* = 9), paired t-test, **P < 0.01. e) CaMPARI2 green and red fluorescence in the *Gr1*+ MD1 glomerulus after stimulation with CO_2_ and synthetic air. Right panels are heatmaps of red fluorescence intensity. MD1 from the left antennal lobe was imaged at 63× magnification. Scale bars: 10 µm. f) CaMPARI2 photoconversion values in MD1, mean *R*/*G* values ± SEM plotted, *n* = 3–5 brains per stimulus, Mann–Whitney U test, *P = 0.037.

To test whether the MD1 glomerulus which receives innervations from *Gr1*+ OSNs responds to CO_2_, we generated mosquitoes suitable for central calcium imaging from the antennal lobe. To do this, we crossed our *Gr1^QF2-MF^* driver and *15xQUAS-GCaMP6s* responder lines to make *Gr1^QF2-MF^* > *30xQUAS-GCaMP6s*. This strain has 1 copy of the *Gr1^QF2-MF^* driver and 2 copies of the *15xQUAS-GCaMP6s* transgene to express GCaMP6s (Chen *et al*. 2013) in the cytoplasm of *Gr1*+ OSNs. Head-tethered *A. aegypti* females of this genotype with surgically exposed antennal lobes bathed in saline were then stimulated with different concentrations of CO_2_. Consistent with observations from peripheral calcium imaging indicating *Gr1*+ neurons are tuned to CO_2_, MD1 exhibited strong increases in odor-evoked GCaMP6s activity in response to stimulation with this gas (Fig. 6a–c). We then quantified the maximum change in GCaMP6s fluorescence intensity in response to stimulation with a CO_2_ concentration series of 0.1, 0.5, and 1% CO_2_ and determined that MD1 exhibits dose-dependent responses at this level of olfactory coding (Fig. 6d).

We also applied the calcium-modulated photoactivatable ratiometric indicator (CaMPARI2) (Moeyaert *et al*. 2018) to record CO_2_ activity from this glomerulus. CaMPARI2 photoconverts from green to red when simultaneously exposed to 405-nm light and high levels of calcium (Fosque *et al*. 2015; Moeyaert *et al*. 2018) and has previously been applied for activity-dependent neural labeling only in other model organisms such as mice, zebrafish, and flies. We generated a *15xQUAS-CaMPARI2* line via Mos1 mariner transposition (Supplementary Table 7 in Supplementary File 1) and then made a genotype for CaMPARI2 imaging with 1 copy of the *Gr1^QF2-MF^* driver 2 copies of this responder transgene (*Gr1^QF-MF^ > 30xQUAS-CaMPARI2*) to express CaMPARI2 in the cytoplasm of *Gr1*+ OSNs. Consistent with GCaMP6s imaging results, MD1 exhibited a significantly higher rate of CaMPARI2 photoconversion in CO_2_-stimulated mosquitoes vs those that were stimulated with synthetic air (Fig. 6e and f), validating the CO_2_ sensitivity of this glomerulus.

These optimized genetic tools therefore facilitate clear imaging of odor-evoked activity from the *A. aegypti* antennal lobe and illustrate that the CO_2_ receptor glomerulus MD1 which receives innervations from *Gr1*+ OSNs is sensitively tuned to carbon dioxide.

## Discussion

The yellow fever mosquito *A. aegypti* is an emerging model system for invertebrate neurobiology. There has been a proliferation of sensory biology studies in this disease vector over the past decade (DeGennaro *et al*. 2013; McBride *et al*. 2014; McMeniman *et al*. 2014; van Breugel *et al*. 2015; Duvall *et al*. 2019; Raji *et al*. 2019; Jové *et al*. 2020; Melo *et al*. 2020; De Obaldia *et al*. 2022; Herre *et al*. 2022; San Alberto *et al*. 2022; Sorrells *et al*. 2022; Zhao *et al*. 2022; Chandel *et al*. 2024; Rouyar *et al*. 2024). To complement these efforts, here we report optimized genetic tools for neuroanatomical and functional mapping of the *A. aegypti* olfactory system. CRISPR–Cas9-mediated HDR has been an extremely important tool to generate *T2A-QF2* in-frame fusions, rapidly expanding the number of cell-type-specific driver lines available in this mosquito species (Matthews *et al*. 2019; Jové *et al*. 2020; Zhao *et al*. 2021, 2022; Herre *et al*. 2022; Laursen *et al*. 2023). However, our study revealed several important issues for consideration when performing these types of transgenic manipulations in *A. aegypti*.

Firstly, we determined that when using circular donor template for CRISPR–Cas9 HDR, this can result in donor cassette integration either via canonical HDR, or alternatively via noncanonical HDR involving duplication of the intended *T2A-QF2* integration cassette separated by intervening donor plasmid sequence. While both integration events yield precise *T2A-QF2* in-frame fusions, duplication of the donor cassette and insertion of intervening vector backbone into the integration site may complicate molecular genotyping and cause other undesired effects. Including a second fluorescent marker in the plasmid backbone outside of the homology arms enabled us to detect this mode of integration. During gene targeting, we only observed noncanonical HDR events for *Gr1*, a single canonical HDR event for *orco*, and a mixture these HDR repair outcomes for *Ir8a*. While it is evident from this study that these differing types of HDR repair outcome are possible across these select target genes, additional studies are required to discern the relative frequency of canonical vs noncanonical HDR when using circular donor template at an expanded number of genetic loci in this mosquito.

We further demonstrated that strategic positioning of loxP sites surrounding the *3xP3* marker in the intended integration cassette can further be used to remove these florescent markers, including the second *T2A-QF2* copy, and any extraneous vector backbone sequence integrated during multiplexed noncanonical HDR events. Evidence of multiple insertions of donor cassettes and plasmid backbone has been also observed in *Drosophila* and human cells (Bier *et al*. 2018; Bandyopadhyay *et al*. 2021; Zirin *et al*. 2022; Loehlin *et al*. 2023), suggesting that this mode of integration may be a common phenomenon when using circular donor template for CRISPR–Cas9-mediated HDR. We therefore highly recommend usage of the above design features where possible when employing circular HDR donor constructs for use with CRISPR–Cas9-mediated HDR in *A. aegypti* or other species.

We initially applied our transgenic reagents for functional imaging from the peripheral nervous system and observed that strong melanization of the *A. aegypti* cuticle dampened CO_2_-evoked GCaMP6s responses in WT females. Introgression of our lines into a *yellow* mutant background (Li *et al*. 2017) allowed for a 2–4-fold increase in the signal recorded from CO_2_-sensitive neurons in the maxillary palps and a larger dynamic range of responses along a CO_2_ dose–response curve. This optimized method will thus facilitate future calcium imaging of odor-evoked activity with other subsets of *A. aegypti* OSNs located on maxillary palps or other sensory appendages in responses to stimulation with varied odorants, including components of the human volatilome (Rankin-Turner and McMeniman 2022). This technique may also facilitate high-throughput screens to determine the ligand tuning dynamics of different subsets of OSNs. Calcium imaging can be carried out in multiple sensilla housing various OSN classes simultaneously within a field of view, as opposed to single sensillum recordings (Afify and Potter 2022). Additionally, the increased signal obtained in *yellow* mutants during calcium imaging recordings will facilitate identification of neurons that show low responses to certain ligands that may be missed if imaging was carried out in a WT background.

Introgression of other driver and responder transgenes into the *A. aegypti yellow* mutant background (Li *et al*. 2017), with its lighter cuticular pigmentation, may increase the accuracy of peripheral cell counts particularly for sparser driver lines, as well as facilitating clear visualization of OSNs during antibody labeling and RNA in situ hybridization (Herre 2023a,b). Finally, application of pigmentation deficient *yellow* mutants has recently been employed in *A. coluzzii* to improve optical accessibility of the cuticle to visualize salivary gland colonization by *Plasmodium berghei* sporozoites (Klug *et al*. 2022), indicating the broad applicability of this approach to facilitate studies in both vector biology and neuroscience. However, as the *yellow* gene has been implicated in other insects in various physiological functions including cuticular pigmentation via production of dopamine melanin (Geyer *et al*. 1986; Wittkopp *et al*. 2002), binding dopamine or other biogenic amines (Xu *et al*. 2011), and egg chorion morphology (Noh *et al*. 2021), these potential factors should be considered by researchers when using *yellow* mutants for experimentation.

During evaluation of these transgenic reagents for central brain imaging, we determined that the *3xP3* markers typically used in insect transgenesis (Berghammer *et al*. 1999) induced spurious background fluorescence in the *A. aegypti* central brain when integrated as part of the *T2A-QF2* in-frame fusion cassette at these 3 target genomic loci. Of note, removing these *3xP3* markers by Cre-loxP-mediated cassette excision (Häcker *et al*. 2017) to generate marker-free *T2A-QF2* driver lines solved this problem. As such, given the widespread use of *3xP3* fluorescent markers for neurogenetic tools in *A. aegypti* (Matthews *et al*. 2019; Jové *et al*. 2020; Zhao *et al*. 2021, 2022; Herre *et al*. 2022; Laursen *et al*. 2023), we suggest that future designs of *T2A-QF2* in-frame fusions in this mosquito species incorporate marker transgenes flanked by loxP sites to facilitate the flexibility to remove them for central neuroanatomical and functional imaging studies. This may be especially important within the context neurogenetic studies that use calcium imaging, optogenetics, and neuronal silencing in the *A. aegypti* nervous system where cell-type specificity of transgene expression and low background signal is crucial.

Using our optimized transgenic tools and immunohistochemistry, we could reliably identify 74 glomeruli in the female *A. aegypti* antennal lobe that were spatially conserved in our in vitro preparations by cross-referencing nc82 and GFP channels to demarcate glomerular boundaries. We determined that *Ir8a*+ OSNs innervate 15 glomeruli mostly localized to the posterolateral antennal lobe region, while *Gr1*+ OSNs innervate a single mediodorsal glomerulus called MD1 (Shankar and McMeniman 2020). We also showed that *orco*+ OSNs innervate a majority of spatially invariant antennal lobe glomeruli (56/74) that we identified and additionally project to the taste center of the mosquito brain known as the SEZ. A similar innervation pattern of *orco*+ OSNs to the SEZ from labellar neurons was observed in *A. coluzzii* (Riabinina *et al*. 2016), where it was hypothesized that this brain region may serve as center for gustatory and olfactory integration. Based on our observations of peripheral innervation of the *A. aegypti* labella by *orco*+ OSNs, future anterograde tracing is required to discern if this peripheral population of labellar neurons also project to the SEZ in this mosquito. Furthermore, whether the SEZ similarly integrates smell and taste information in *A. aegypti* remains unexplored. Finally, we estimate that a small subset of glomeruli in the *A. aegypti* antennal lobe co-express *Ir8a* and *orco* based on overlapping patterns of glomerular labeling in this brain center, as antennal immunostaining experiments with complementary *T2A-QF2* driver reagents developed by others previously suggested (Herre *et al*. 2022).

Reduced or more variable numbers of antennal lobe glomeruli and receptor-to-glomerulus labeling patterns using *T2A-QF2* in-frame fusions in *A. aegypti* have been recently described by 2 complementary studies, which found ∼60 (Zhao *et al*. 2022) or 60–72 total glomeruli (Herre *et al*. 2022). The upper range of these estimates is concordant with the 74 spatially invariant glomeruli that we observed in this study. We find that the use of our marker-free *T2A-QF2* driver strains devoid of background interference from *3xP3* fluorescent markers, and a cumulative 30 copies of membrane-tethered GFP with translational enhancers (Pfeiffer *et al*. 2012) to strongly label OSNs, facilitates a clearer delineation of glomerular boundaries within the *A. aegypti* antennal lobe. As the repertoire of cell-type-specific driver lines for *A. aegypti* chemoreceptors increases, the annotation of glomeruli detailed here may be further refined. Indeed, the design principles described here to generate marker-free *T2A-QF2* drivers devoid of background interference from transgenesis markers will be useful toward that end.

Moving forward, systematic receptor-to-glomerulus mapping in *A. aegypti* will help to discern whether certain glomeruli adjacent to one another represent different glomeruli or alternatively fragmented arborizations of the same glomerulus. Such morphological distortions may explain differences in the number of glomeruli observed across in vitro studies. Since additional improvements toward understanding the stereotypy of this brain region will lead to changes in glomerular numbering and naming over time as observed iteratively for *Drosophila melanogaster* (Stocker *et al*. 1990; Laissue *et al*. 1999; Couto *et al*. 2005; Fishilevich and Vosshall 2005; Tanaka *et al*. 2012; Grabe *et al*. 2015; Task *et al*. 2022), we have included raw confocal stacks and annotation files (Supplementary data) to facilitate future supplementary analysis or reannotation by the community.

We confirmed that the CO_2_ receptor glomerulus MD1 is the largest neuropil in the *A. aegypti* antennal lobe (Ignell *et al*. 2005; Shankar and McMeniman 2020). The large volume of MD1 may indicate extensive synaptic connectivity between CO_2_ receptor neurons, local interneurons, and projection neurons innervating higher-order olfactory processing centers of the mosquito brain. This likely reflects the critical importance of CO_2_ to multiple facets of *A. aegypti* host-seeking behavior (Carlson *et al*. 1973; Geier *et al*. 1999; Bernier *et al*. 2003; Dekker *et al*. 2005; Dekker and Cardé 2011; McMeniman *et al*. 2014; Vinauger *et al*. 2019, #33; Sorrells *et al*. 2022; Chandel *et al*. 2024). We also reported for the first time the use of the calcium-modulated photoactivatable ratiometric indicator (CaMPARI2) (Moeyaert *et al*. 2018) in a nonconventional model organism. While CaMPARI2 cannot be used to record real-time neuronal activity like GCaMP indicators, it still provides a useful tool to study the activation of glomeruli deep in the antennal lobe where live imaging with GCaMP may be challenging and strongly affected by movement artifacts. Alternatively, this sensor may have utility for activity-dependent labeling in other brain regions in conjunction with other cell-type-specific or pan-neuronal drivers developed for *A. aegypti* (Matthews *et al*. 2019; Jové *et al*. 2020; Zhao *et al*. 2021, 2022; Herre *et al*. 2022; Laursen *et al*. 2023).

In this study, we generated disruptive *T2A-QF2* in-frame fusions in coding exons of *orco*, *Ir8a*, and *Gr1* and demonstrated the utility of these drivers when used as heterozygotes for functional imaging and neuroanatomy. However, it is worth noting that when using these drivers as homozygotes that loss-of-function phenotypes in these olfactory genes may be observed. When expanding this method to additional target loci, such as those implicated in development or physiology, careful consideration of whether the target gene is haplosufficient for the phenotype of interest is required. In such cases, *T2A-QF2* in-frame fusions can be directed toward to the C terminus of the endogenous coding region in the target gene to reduce chances of gene disruption (Zhao *et al*. 2021; Herre *et al*. 2022).

The optimized genetic tools we report here may therefore be highly useful to study the molecular and cellular basis of *A. aegypti* olfaction including identification of chemosensory circuitry that mediates attraction to humans, oviposition sites, and mates. High-resolution studies of mosquito olfactory biology using neurogenetics may reveal rational targets for development of interventions to combat mosquito-borne diseases transmitted by *A. aegypti* such as dengue, Zika, and chikungunya.

## Supplementary Material

jkae307_Supplementary_Data

## Data Availability

Strains and plasmids are available upon request. The authors affirm that all data necessary for confirming the conclusions of the article are present within the article, figures, and supplementary material. All raw data supporting these analyses have been deposited publicly in the Johns Hopkins Research Data Repository at: https://doi.org/10.7281/T1/HSROGO. The full sequences of plasmids generated in this study have been deposited to GenBank: *pBB-AaGr1* (accession no. PQ659180), *pBB-AaOrco* (accession no. PQ671723), *pBB-AaIR8a* (accession no. PQ671724), *pBB* (accession no. PQ671725), *pMos-loxP-ECFP-loxP-15xQUAS-CaMPARI2* (accession no. PQ671726), *pMosECFP-15xQUAS-GCaMP6s* (accession no. PQ671727), *pMosECFP-15xQUAS-mCD8GFP* (accession no. PQ671728), and *pMosEYFP-exu-Cre* (accession no. PQ671729). Supplemental material available at G3 online.
