# Supplementary material for: Optimized genetic tools for neuroanatomical and functional mapping of the Aedes aegypti olfactory system
Source: G3 (Bethesda). 2025 Jan 24;15(3):jkae307. doi: 10.1093/g3journal/jkae307 (PMC11917485; doi:10.1093/g3journal/jkae307)
Supplement: jkae307_Supplementary_Data [file jkae307_supplementary_data.pdf]

SUPPLEMENTAL MATERIAL

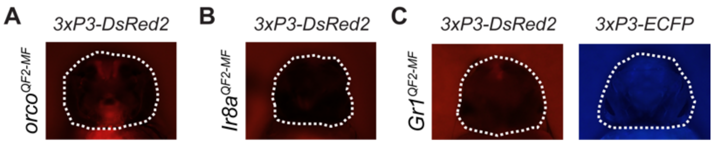

Figure S1. Absence of *3xP3* marker expression in Cre-loxP excised marker-free driver lines. Fourth instar larvae from **(A)** $orco^{QF2\text{-}MF}$, **(B)** $Ir8a^{QF2\text{-}MF}$ and **(C)** $Gr1^{QF2\text{-}MF}$ driver lines. Heads outlined with dashed lines.

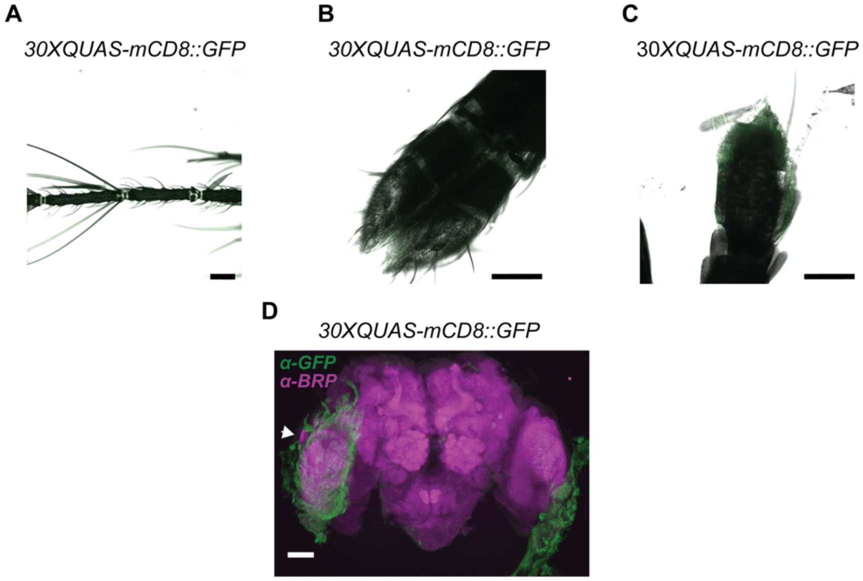

Figure S2. Lack of background GFP fluorescence in peripheral sensory appendages and antennal lobes from the *30xQUAS-mCD8::GFP* responder only control line. No apparent background GFP fluorescence derived from olfactory sensory neurons is observed in adult female peripheral sensory appendages including the **(A)** antenna, **(B)** labella **(C)** maxillary palp, as well as the adult female **(D)** antennal lobe from the *30xQUAS-mCD8::GFP* responder only control line. Arrows indicate expression of the *3xP3-ECFP* transgenesis marker for the *QUAS-mCD8::GFP* responder transgene in the outer optic lobes. Maximum intensity projections are shown. Scale bars = 50μm.

orco$^{QF2\text{-}MF}$ > *30XQUAS-mCD8::GFP*

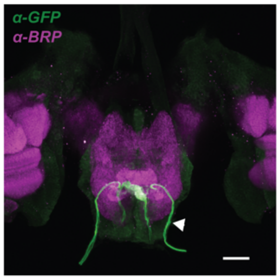

Figure S3. SEZ innervation by *orco*+ neurons. Posterior view of the brain of an adult female *orco*$^{QF2\text{-}MF}$ > *30xQUAS-mCD8::GFP* mosquito. *orco*+ neurons from the labella project via the labial nerve (arrow) and terminate in the gustatory center of the mosquito brain, the subesophageal zone (SEZ). Scale bar: 40 μm.

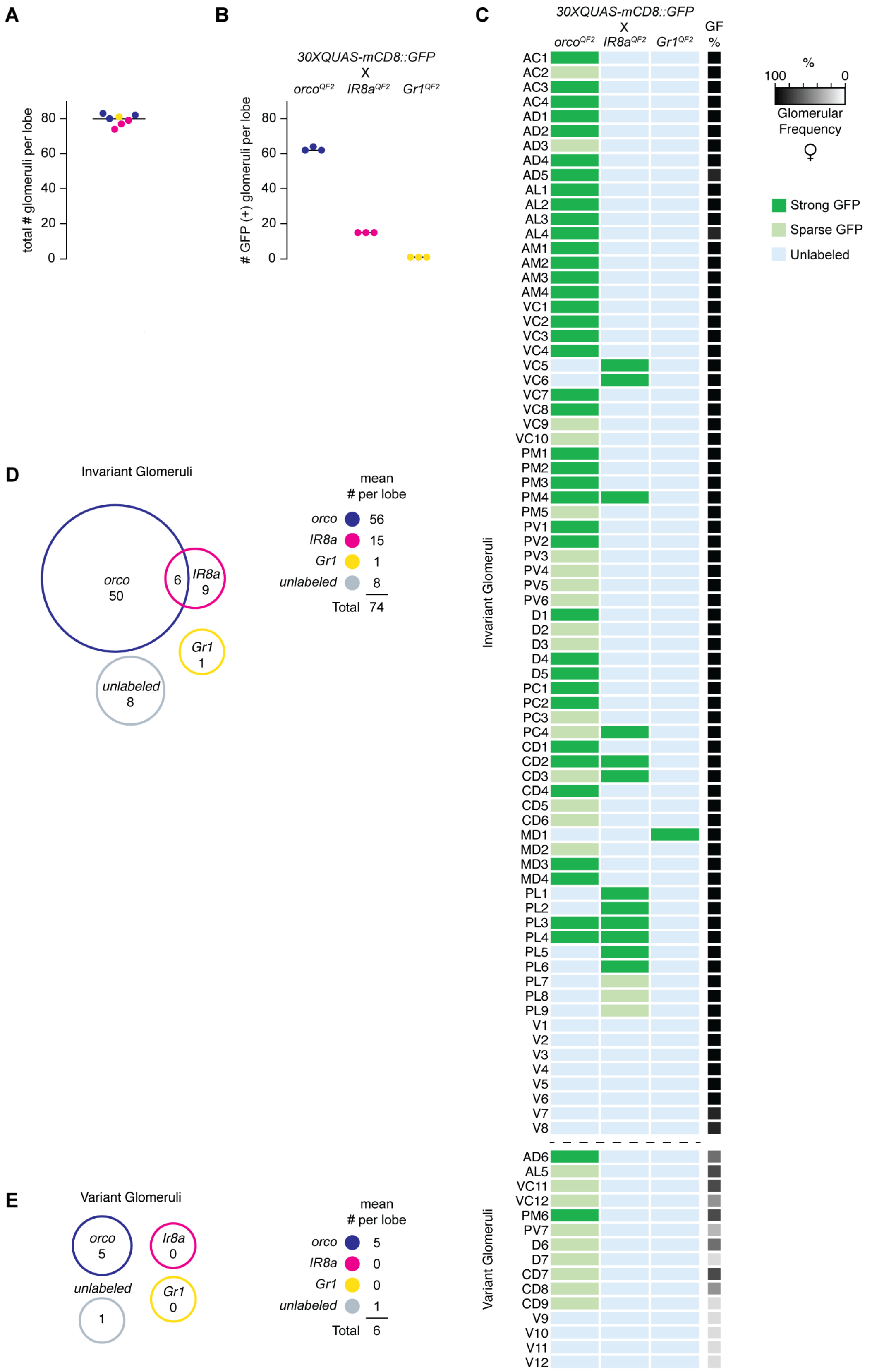

A
total # glomeruli per lobe
0
20
40
60
80
B
30XQUAS-mCD8::GFP
X
orco^QF2
IR8a^QF2
Gr1^QF2
GFP (+) glomeruli per lobe
0
20
40
60
80
C
30XQUAS-mCD8::GFP
X
orco^QF2
IR8a^QF2
Gr1^QF2
GF %
%
100
0
Glomerular Frequency
♀
Strong GFP
Sparse GFP
Unlabeled
Invariant Glomeruli
AC1 AC2 AC3 AC4 AD1 AD2 AD3 AD4 AD5 AL1 AL2 AL3 AL4 AM1 AM2 AM3 AM4 VC1 VC2 VC3 VC4 VC5 VC6 VC7 VC8 VC9 VC10 PM1 PM2 PM3 PM4 PM5 PV1 PV2 PV3 PV4 PV5 PV6 D1 D2 D3 D4 D5 PC1 PC2 PC3 PC4 CD1 CD2 CD3 CD4 CD5 CD6 MD1 MD2 MD3 MD4 PL1 PL2 PL3 PL4 PL5 PL6 PL7 PL8 PL9 V1 V2 V3 V4 V5 V6 V7 V8
Variant Glomeruli
AD6 AL5 VC11 VC12 PM6 PV7 D6 D7 CD7 CD8 CD9 V9 V10 V11 V12
D
Invariant Glomeruli
orco
50
6
IR8a
9
Gr1
1
unlabeled
8
mean
per lobe
orco 56
IR8a 15
Gr1 1
unlabeled 8
Total 74
E
Variant Glomeruli
orco
5
Ir8a
0
unlabeled
1
Gr1
0
mean
per lobe
orco 5
IR8a 0
Gr1 0
unlabeled 1
Total 6

Figure S4. Classification and frequency of *Aedes aegypti* antennal lobe glomeruli. (A) Total number of glomeruli per reconstructed antennal lobe (n = 7). **(B)** Number of GFP labeled glomeruli per genotype (n = 3). **(C)** Innervation pattern of GFP-labeled *orco*, *Ir8a* and *Gr1*+ neurons in the *Ae. aegypti* antennal lobe. Glomeruli were either strongly or sparsely labeled with mCD8::GFP, or unlabeled. A heatmap of glomerular frequency (GF %) is shown in parallel. 74 spatially invariant glomeruli were characterized across reconstructions (frequency ≥ 80%). 15 variant glomeruli with different annotated names were also found across some reconstructions (frequency < 80%). **(D** to **E)** Venn diagrams (left) summarizing the mean number of invariant and variant glomeruli per lobe annotated as being *orco*+, *Ir8a*+, *orco*+:*Ir8a*+, *Gr1*+ or unlabeled using the reference atlas. The mean number of cumulative glomeruli putatively innervated by each chemoreceptor class (right) is shown.

Table S1. Complete genotypes of *Aedes aegypti* stocks and composite genotypes used in this study.

Identifier	ABBREVIATED GENOTYPE	Marker (s)	FULL GENOTYPE<br>***Aedes aegypti* chromosome 1; 2; 3**<br>+ = wild-type allele
Stock	*LVPib12*	*none - wild-type*	*+/+; +/+; +/+*
Stock	*yellow mutant:* y^{1}	*yellow cuticle*	y^{1} / y^{1}*; +/+; +/+*
Stock	*Gr1*$^{QF2\text{-}3xP3}$	*3xP3-DsRed2, 3xP3-ECFP, 3xP3-DsRed2*	*+/+; Gr1*$^{QF2\text{-}3xP3}$ */ Gr1*$^{QF2\text{-}3xP3}$*; +/+*
Stock	*Ir8a*$^{QF2\text{-}3xP3}$	*3xP3-DsRed2*	*Ir8a*$^{QF2\text{-}3xP3}$ */ Ir8a*$^{QF2\text{-}3xP3}$*; +/+; +/+*
Stock	*orco*$^{QF2\text{-}3xP3}$	*3xP3-DsRed2*	*+/+; +/+; orco*$^{QF2\text{-}3xP3}$ */ +*
Stock	*Gr1*$^{QF2\text{-}MF}$	*marker-free*	*+/+; Gr1*$^{QF2\text{-}MF}$ */ Gr1*$^{QF2\text{-}MF}$*; +/+*
Stock	*Ir8a*$^{QF2\text{-}MF}$	*marker-free*	*Ir8a*$^{QF2\text{-}MF}$ */ Ir8a*$^{QF2\text{-}MF}$*; +/+; +/+*
Stock	*orco*$^{QF2\text{-}MF}$	*marker-free*	*+/+; +/+; orco*$^{QF2\text{-}MF}$ */ +*
Stock	*15xQUAS-mCD8::GFP*	*3xP3-ECFP*	*+/+; 15xQUAS-mCD8::GFP / 15xQUAS-mCD8::GFP; +/+*
Stock	*15xQUAS-CaMPARI2*	*3xP3-ECFP*	*15xQUAS-CaMPARI2 / 15xQUAS-CaMPARI2; +/+; +/+*
Stock	*15xQUAS-GCaMP6s*	*3xP3-ECFP*	*15xQUAS-GCaMP6s / 15xQUAS-GCaMP6s; +/+; +/+*
Stock	*15xQUAS-GCaMP6s [*y^{1}*]*	*3xP3-ECFP (yellow cuticle)*	y^{1}*, 15xQUAS-GCaMP6s /* y^{1}*, 15xQUAS-GCaMP6s; + / +; +/+*
Stock	*Gr1*$^{QF2\text{-}3xP3}$ *[*y^{1}*]*	*3xP3-DsRed2, 3xP3-ECFP, 3xP3-DsRed2 (yellow cuticle)*	y^{1} */* y^{1}*; Gr1*$^{QF2\text{-}3xP3}$ */ +; +/+*
Stock	*exu-Cre*	*PUb-EYFP*	*+/+; exu-Cre / +; +/+*
Composite	*Gr1*$^{QF2\text{-}3xP3}$ *> 15xQUAS-mCD8::GFP*	*3xP3-DsRed2, 3xP3-ECFP, 3xP3-DsRed2 + 3xP3-ECFP*	*+/+; Gr1*$^{QF2\text{-}3xP3}$ */ 15xQUAS-mCD8::GFP; +/+*
Composite	*orco*$^{QF2\text{-}3xP3}$ *> 15xQUAS-mCD8::GFP*	*3xP3-DsRed2 + 3xP3-ECFP*	*+/+; 15xQUAS-mCD8::GFP / +; orco*$^{QF2\text{-}3xP3}$ */ +*
Composite	*Ir8a*$^{QF2\text{-}3xP3}$ *> 15xQUAS-mCD8::GFP*	*3xP3-DsRed2 + 3xP3-ECFP*	*Ir8a*$^{QF2\text{-}3xP3}$ */ +; 15xQUAS-mCD8::GFP / +; +/+*
Composite	*Gr1*$^{QF2\text{-}3xP3}$ *[*y^{1}*] > 15xQUAS-GCaMP6s [*y^{1}*]*	*3xP3-DsRed2, 3xP3-ECFP, 3xP3-DsRed2 (yellow cuticle) + 3xP3-ECFP (yellow cuticle)*	y^{1}*, 15xQUAS-GCaMP6s /* y^{1}*; Gr1*$^{QF2\text{-}3xP3}$ */ +; +/+*
Composite	*Gr1*$^{QF2\text{-}MF}$ *> 30xQUAS-mCD8::GFP*	*marker-free + 3xP3-ECFP*	*+/+; Gr1*$^{QF2\text{-}MF}$*, 15xQUAS-mCD8::GFP / 15xQUAS-mCD8::GFP; +/+*
Composite	*orco*$^{QF2\text{-}MF}$ *> 30xQUAS-mCD8::GFP*	*marker-free + 3xP3-ECFP*	*+/+; 15xQUAS-mCD8::GFP / 15xQUAS-mCD8::GFP; orco*$^{QF2\text{-}MF}$ */ +*
Composite	*Ir8a*$^{QF2\text{-}MF}$ *> 30xQUAS-mCD8::GFP*	*marker-free + 3xP3-ECFP*	*Ir8a*$^{QF2\text{-}MF}$ */ +; 15xQUAS-mCD8::GFP / 15xQUAS-mCD8::GFP; +/+*
Composite	*Gr1*$^{QF2\text{-}MF}$ *> 30xQUAS-GCaMP6s*	*marker-free + 3xP3-ECFP*	*15xQUAS-GCaMP6s / 15xQUAS-GCaMP6s; Gr1*$^{QF2\text{-}MF}$ */ +; +/+*
Composite	*Gr1*$^{QF2\text{-}MF}$ *> 30xQUAS-CaMPARI2*	*marker-free + 3xP3-ECFP*	*15xQUAS-CaMPARI2 / 15xQUAS-CaMPARI2; Gr1*$^{QF2\text{-}MF}$ */ +; +/+*

Table S2. CRISPR target sites and locations.

**Gene**	**Identifier**	**Chromosome**	**Band**	**CRISPR target site** (with **NGG**)	**Cut Site**
orco	AAEL005776	3	3p22	CCATCAAGGCTTGGTACCCG **TGG**	Exon 3
Ir8a	AAEL002922	1	1p22	TCCAACGCGAAAGTACCGCT **TGG**	Exon 3
Gr1	AAEL002380	2	2q23	TGACCATGAGGTACTTATAC **TGG**	Exon 3

Table S3. Primers for amplifying homology arms for *T2A-QF2* donor constructs.

Gene region	Homology Arm Size (bp)	Homology Arm Primers with In-Fusion Adapters (underlined)
Gr1 Left Arm	1003	5’-TATAACCCGCCTCGGTTATTCCGTTTGTTTTCAATTTTCCGCG-3’<br>5’-TGCCGCGGCCCTCTCCGCTTCCCTGGTAGTCGGTCCACATGT-3’
Gr1 Right Arm	884	5’-TGACAGATCTGCGCGTAAGTACCTCATGGTCACCGGA-3’<br>5’-GATATCGATCGCGCGACCGTTTGGAGGTTGAATTG-3’
orco Left Arm	2624	5’-TATAACCCGCCTCGGTGCAAGTGGATCATTTGTCG-3’<br>5’-TGCCGCGGCCCTCTCCGCTTCCGTACCAAGCCTTGATGGGC-3’
orco Right Arm	1329	5’-TGACAGATCTGCGCGCCGTGGGATGCAATGAG-3’<br>5’-GATATCGATCGCGCGGTGCAATTGTGCCATTTTGA-3’
Ir8a Left Arm	2159	5’-TATAACCCGCCTCGGCAAAGTATAATTTCGCCCCCTCC-3’<br>5’-TGCCGCGGCCCTCTCCGCTTCCCTTGGTCGGTTTGATCTTCTG-3’
Ir8a Right Arm	1964	5’-TGACAGATCTGCGCGGGTACTTTCGCGTTGGAACTA-3’<br>5’-GATATCGATCGCGCGCTCTATGGCAGCCAAGATATTGG-3’

Table S4. Template materials for constructs.

**Plasmid**	**Element**	**Source**		**Addgene #**
pBB				
	3xP3-ECFP-SV40	pBAC-ECFP-15xQUAS-TATA-SV40		104875
	T2A-QF2-hsp70-loxP	pHACK-QF2		80274
	3xP3 promoter	pBAC-ECFP-15xQUAS-TATA-SV40		104875
	DsRed2-SV40	pMOS-3xP3-DsRed		n/a
All responder constructs				
	Mos1 and 3xP3-ECFP marker	pMos{3xP3-ECFPaf}		n/a
	15xQUAS promoter	pBAC-ECFP-15xQUAS-TATA-SV40		104875
	Syn21 enhancer	pJFRC81-10XUAS-IVS-Syn21-GFP-p10		36432
	p10 terminator	pJFRC81-10XUAS-IVS-Syn21-GFP-p10		36432
pMosECFP-15xQUAS-mCD8-GFP	mCD8:GFP	pQUASp-mCD8::GFP		46163
pMosECFP-15xQUAS-GCaMP6s	GCaMP6s	pGP-CMV-GCaMP6s		40753
pMos-loxP-ECFP-loxP-15xQUAS-CaMPARI2				
	CaMPARI2	pAAV-hsyn1-CaMPARI2	gift of E. Schreiter	
pMosEYFP-exu-Cre				
	Mos1	pMOS-3xP3-dsRed		n/a
	Polyubiquitin promoter	pSL1180-HR-PUbECFP		47917
	EYFP	pBAC-YFP-QF2-hsp70	gift of C. Potter	
	exuperantia promoter	*AAEL010097-Cas9* mosquitoes	gift of O. Akbari	
	Cre recombinase	pENTR L1-vas2-Cre-L4		62301
	p10 terminator	pJFRC81-10XUAS-IVS-Syn21-GFP-p10		36432

Table S5. Genomic integration sites of *Mos1 mariner* transgenes.

Transgene	Chromosome	Band	Insertion Site	Insertion Location
15xQUAS-CaMPARI2	1	1p25	65092968	AAEL026960, Intron
15xQUAS-mCD8::GFP	2	2q24	329084910	Upstream of AAEL021072 lncRNA
15xQUAS-GCaMP6s	1	1q44	301298340	Intergenic region between AAEL010596 and AAEL010590
exu-Cre	2	2q24	324350134	AAEL013635, Intron

Table S6. Microinjection statistics for *T2A-QF2* HDR constructs.

HDR Construct	Cas9 + gRNA delivery format	Embryos Injected	% hatch	G_0 adult survivors		G_1 transgenics isolated per mass cross [d]	
				female	male	G_0 female x *LVPib12*	*LVPib12* x G_0 male
pBB-AaGr1	Cas9 recombinant protein + gRNA > *LVPib12* [a]	1615	57	407	387	2 (DsRed2, ECFP)	0
pBB-AaIR8a	gRNA > *exu-Cas9* [b]	655	30	82	95	12 (DsRed2)<br>5 (DsRed2, ECFP)	0
pBB-AaOrco	gRNA > *exu-Cas9* [b]	815	> 40 [c]	171	158	1 (DsRed2)	0

[a] Injected by UMD-ITF into the *LVPib12 Aedes aegypti* strain, gRNA in vitro transcribed.

[b] Injected by JHU (McMeniman laboratory) into the *Aedes aegypti exu-Cas9* strain, gRNA in vitro transcribed.

[c] Exact hatch rate not recorded. Estimated % hatch = G_0 adult survivors/embryos injected.

[d] Number of *3xP3-DsRed2* or *3xP3-DsRed2*/*3xP3-ECFP* positive G_1 larvae isolated per mass cross.

Table S7. Microinjection statistics for *QUAS* responder *Mos1 mariner* constructs.

***Mos1 mariner* Construct** [a]	**Embryos Injected**	**% hatch**	**G_0 adult survivors**		**G_1 transgenics isolated per mass cross** [b]	
			**female**	**male**	**G_0 female x *LVPib12***	***LVPib12* x G_0 male**
pMosECFP-15xQUAS-mCD8GFP	534	57	146	149	0	10
pMosECFP-15xQUAS-GCaMP6s	561	12	23	21	0	25
pMosEYFP-exu-Cre	1033	32	47	106	0	1
pMos-loxP-ECFP-loxP-15xQUAS-CaMPARI2	1303	29	127	138	0	6

[a] All *Mos1 mariner* constructs were co-injected with pKhsp82 transposase helper plasmid by UMD-ITF into the *LVPib12 Aedes aegypti* strain.

[b] Number of *3xP3-ECFP* positive G_1 larvae isolated per mass cross.
